# BNIP3‐Dependent Mitophagy Non‐Autonomously Regulates Systemic Aging via NF‐κB Suppression in *Drosophila*

**DOI:** 10.1111/acel.70539

**Published:** 2026-05-13

**Authors:** Zhouyang Deng, Hailong Han, Caifang Wang, Wen Zhong, Zhengqing Wan, Dan Zhou, Ye Chen, Yanni Peng, Zongzhao Zhai, Kai Yuan, Ruoxi Wang, Zhuohua Zhang

**Affiliations:** ^1^ Institute of Molecular Precision Medicine and Hunan Provincial Key Laboratory of Molecular Precision Medicine Xiangya Hospital, Central South University Changsha Hunan China; ^2^ Hunan Provincial Key Laboratory of Medical Genetics, College of Biological Sciences Central South University Changsha Hunan China; ^3^ Department of Neurosciences, Hengyang Medical School University of South China Hengyang Hunan China; ^4^ Institute of Cytology and Genetics, School of Basic Medical Sciences, Hengyang Medical School University of South China Hengyang Hunan China; ^5^ Department of Medical Genetics Hunan Provincial Maternal and Child Health Care Hospital Changsha Hunan China; ^6^ Hunan Provincial Key Laboratory of Animal Intestinal Function and Regulation, College of Life Sciences Hunan Normal University Changsha Hunan China; ^7^ Department of Neurosciences, School of Life Sciences Southern University of Science and Technology Shenzhen Guangdong China

**Keywords:** aging, BNIP3, inflammation, mitophagy, neurodegeneration, non‐autonomous regulation

## Abstract

Aging is a major risk factor for numerous diseases, including degenerative and metabolic disorders. Cumulative mitochondrial damage, elevated reactive oxygen species (ROS), and impaired mitophagy are hallmarks of aging. In this study, we generated a *Drosophila* version of the mito‐SRAI reporter to monitor mitophagy in vivo and demonstrated an age‐dependent decline in muscle mitophagy, accompanied by the accumulation of insoluble proteins, increased ROS levels, and mitochondrial damage. Overexpression of *BNIP3* preserved muscle homeostasis by enhancing mitophagy, maintaining mitochondrial integrity, and suppressing ROS accumulation. Importantly, muscle‐specific expression of *BNIP3* in indirect flight muscles extended lifespan and alleviated age‐associated neurodegenerative phenotypes, including protein aggregation, β‐galactosidase accumulation, and pathological vacuolization in the brain. Mechanistically, BNIP3 inhibited ROS‐mediated activation of Relish, thereby reducing expression of antimicrobial peptide (AMP) genes. These findings identify BNIP3 as a key regulator of aging that links mitochondrial quality control to systemic aging and neurodegeneration. Moreover, our results provide direct evidence of muscle‐to‐brain signaling, revealing a non‐autonomous mechanism by which muscle mitophagy mitigates age‐related neurodegeneration.

## Graphical abstract

Muscle aging is characterized by alterations in mitochondrial morphology and structure, leading to the accumulation of reactive oxygen species (ROS). This increase in ROS activates Key/Relish‐mediated inflammatory pathways, resulting in the production of antimicrobial peptides (AMPs) that further accelerate aging. BNIP3‐mediated mitophagy in muscle regulates ROS production to modulate the activation of inflammatory pathways so that it maintains muscle homeostasis and delays systemic aging.

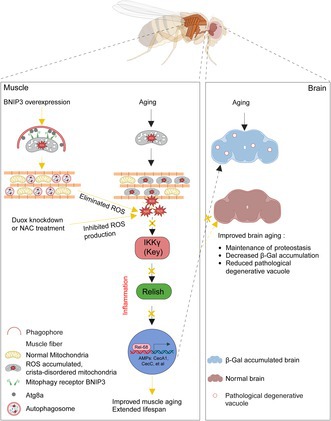

## 1 Introduction

Aging is a complex biological process characterized by a progressive decline in physiological functions across multiple organ systems, leading to increased susceptibility to diseases and mortality (Guo et al. 2022; Lopez‐Otin et al. 2023). Skeletal muscle, the largest tissue by mass in the human body, plays essential roles in posture, movement, respiration, and metabolism (Handschin et al. 2007; Merz and Thurmond 2020). In addition, skeletal muscle is increasingly recognized as an active regulator of systemic aging rather than a passive tissue affected by age (Kedlian et al. 2024; Lai, Ramirez‐Pardo, et al. 2024). Through its endocrine, metabolic, and regenerative functions, particularly via the secretion of signaling molecules known as myokines, it exerts wide‐ranging effects on other tissues (Barros et al. 2022; Hoffmann and Weigert 2017). However, the molecular and cellular mechanisms by which skeletal muscle influences the aging process, and how muscle senescence contributes to degenerative changes in distant organs, remain unclear.

Mitochondria are essential for skeletal muscle function due to the high energy demands of the tissue (Lei et al. 2024). During aging, mitochondrial decline contributes significantly to sarcopenia, fatigue, and metabolic dysregulation (Gherardi et al. 2025). Aging skeletal muscle exhibits mitochondrial dysfunction, impaired biogenesis, increased oxidative stress, and defects in mitochondrial quality control, all of which damage proteins, lipids, and nucleic acids (Al Rawi et al. 2011; Henrich et al. 2023; Zhou et al. 2010). Mitochondrial turnover is particularly critical in muscle because of the constant need for energy during contraction and repair. When mitophagy, the selective degradation of damaged mitochondria, is impaired, dysfunctional mitochondria accumulate and produce excessive reactive oxygen species (ROS), further exacerbating cellular damage (Onishi et al. 2021). This self‐perpetuating cycle of oxidative stress and mitochondrial dysfunction is considered a key driver of muscle senescence (Gouspillou et al. 2014; Kim et al. 2022; Short et al. 2005).

Mitophagy in skeletal muscle is mediated primarily through two pathways, including the PINK1–Parkin pathway and intrinsic mitochondrial membrane proteins such as BNIP3 and FUNDC1 (Irazoki et al. 2022; Sliter et al. 2018; Sun et al. 2018). Among these, BNIP3 plays a pivotal role in stress‐ and aging‐induced mitophagy. While BNIP3 protects against mitochondrial damage during inflammation, its chronic upregulation can promote muscle wasting (Irazoki et al. 2022). Conversely, age‐related reductions in BNIP3 expression impair mitochondrial clearance and contribute to the buildup of senescent cells (Irazoki et al. 2022; Schmid et al. 2022). In *Drosophila*, BNIP3 expression suppresses mitochondrial abnormalities caused by PINK1 inactivation, suggesting that BNIP3 enhancement may counteract certain aging phenotypes (Zhang et al. 2016). Skeletal muscle can also be a source of toxic protein aggregates, metabolic dysregulation, and inflammatory signals that exacerbate central nervous system (CNS) pathology (Demontis and Perrimon 2010; Rai et al. 2021). Clinical observations and studies in model organisms consistently support a systemic role for skeletal muscle in modulating neurodegeneration and organismal aging (Matthews et al. 2023). As a secretory organ, skeletal muscle releases a wide range of bioactive molecules, including myokines, short peptides, and small‐molecule metabolites. Many of them are closely linked to skeletal muscle health and aging (Pedersen 2019; Pedersen and Febbraio 2012). Myokines such as Sestrin1, Sestrin2, Irisin, and VEGF are found present at lower levels in the serum of elderly individuals compared to young adults (Kwon et al. 2020; McCormick et al. 2022; Rajan et al. 2021; Ryan et al. 2006). In addition, stress‐induced amylase and maltose regulate muscle‐to‐CNS signaling, helping to maintain proteostasis in aging brain and retina through chaperone activity (Rai et al. 2021). Despite these advances, the mechanisms through which skeletal muscle shapes organismal aging through inter‐organ communication remain largely unknown.

In this study, we used *Drosophila* as a model to investigate mitophagy in muscle tissues and its regulation of systemic aging. We first developed a novel in vivo tool to monitor mitophagy in *Drosophila* using mito‐SRAI. Using this system, we demonstrated that muscle aging in *Drosophila* is associated with a progressive decline in mitophagy. This decline activates Relish (NF‐κB) mediated inflammatory signaling, which contributes to systemic aging and age‐dependent neurodegeneration. Furthermore, muscle‐specific overexpression of *BNIP3* suppressed age‐associated muscle functional decline and extended lifespan of *Drosophila* through a mitophagy‐dependent mechanism.

## 2 Results

### 2.1 Mito‐SRAI Detects Mitophagy in *Drosophila*

To analyze mitophagy more effectively in vivo, we generated GAL4/UAS‐inducible transgenic fly lines expressing mito‐SRAI, a mitochondria‐targeted mitophagy sensor. Mito‐SRAI consists of TOLLES and YPet, forming a single‐excitation, dual‐emission reporter (Katayama et al. 2020). Upon mitochondrial delivery to lysosomes, YPet undergoes degradation, while TOLLES remains stable. Consequently, the appearance of TOLLES‐only puncta, reflected by an increased TOLLES/YPet fluorescence ratio, indicates mitochondria undergoing lysosomal degradation (Figure S1A).

Flies expressing mito‐SRAI exhibited distinct mitophagy patterns across multiple *Drosophila* tissues. Mitophagy was prominently observed in fly intestine 2 h after puparium formation (2 h APF) (Figure S1B) and in the fat body tissues of third‐instar larvae (L3) following a 4 h starvation (Figure S1C). Muscle‐specific (Mhc‐Gal4) or testis‐specific (Bam‐Gal4) induction of mito‐SRAI expression enabled detection of basal mitophagy in indirect fly muscles (IFMs) and testes, respectively (Figure S1D,E). In fat body cells, mito‐SRAI puncta corresponding to autophagolysosomes were colocalized with Lysotracker Deep Red staining (Figure S1F,G). Furthermore, mitophagy detected by mito‐SRAI was suppressed by RNAi knockdown of core autophagy genes (*atg1*, *atg5*, *atg7*, and *atg12*) during intestinal development at 2 h APF (Figure S1H,I), confirming that mito‐SRAI reliably reports mitophagy in vivo in *Drosophila*.

Mito‐QC and mito‐Keima, both mitochondria‐targeted reporters, have been widely used to detect mitophagy in *Drosophila* (Cornelissen et al. 2018; McWilliams et al. 2018). We next compared the two established reporters with mito‐SRAI for mitophagy detection in vivo. Mito‐SRAI expression effectively monitored lysosome‐mediated mitochondrial degradation, comparable to mito‐QC in fat bodies (Figure S2A), enterocyte cells (Figure S2C), and IFMs (Figure S2E). Similarly, mito‐SRAI also detected mitophagy in living *Drosophila* tissues with comparable sensitivity to mito‐Keima as shown in fat bodies (Figure S2B), intestines (Figure S2D), and IFMs (Figure S2F). Together, these results demonstrate that mito‐SRAI is a robust and sensitive mitophagy reporter under both fixed and live conditions.

### 2.2 Age‐Dependent Decline of Mitophagy and Mitochondrial Metabolism in *Drosophila* IFMs

**FIGURE 1:**
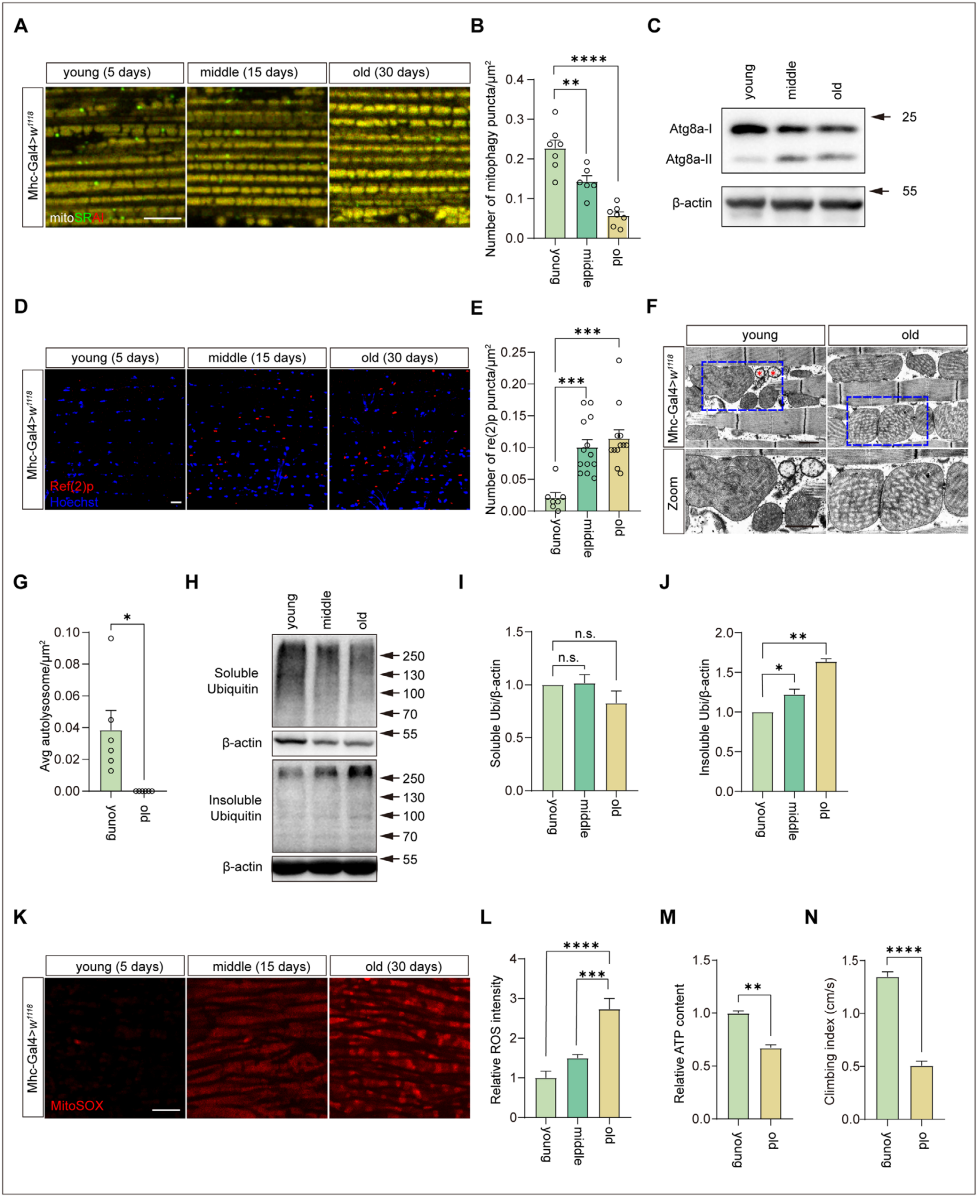
Mitophagy detection using Mito‐SRAI in *Drosophila* with aging. (A, B) Mito‐SRAI expression in IFMs from young (5‐day‐old), middle (15‐day‐old) and old (30‐day‐old) wildtype flies (Mhc‐Gal4>*w* ^*1118*^). Bar = 10 μm (A). Quantification of mito‐SRAI labeled mitophagy. *n* = 7 (young), *n* = 6 (middle), *n* = 7 (old) (B). One‐way ANOVA followed by Tukey's test. ****p < 0.0001, **p < 0.01. Data are from at least three biological replicates and presented as mean ± SEM. (C) Immunoblotting detection of ATG8a in thoraces of young (5‐day‐old), middle (15‐day‐old), and old (30‐day‐old) flies. (D, E) Immunostaining of IFMs from young (5‐day‐old), middle (15‐day‐old) and old (30‐day‐old) wildtype flies (Mhc‐Gal4>*w* ^*1118*^) showing Ref(2)p (red, P62) and nuclei (blue). Bar = 10 μm (D). Quantification of Ref(2)p puncta in IFMs. *n* = 7 (young), 13 (middle), and 12 (old) (E). One‐way ANOVA followed by Tukey's test. ***p < 0.001. Data are from at least three biological replicates and presented as mean ± SEM. (F, G) TEM images IFMs of young (5‐day‐old) and old (30‐day‐old) wildtype flies (Mhc‐Gal4>*w* ^*1118*^). Red asterisks indicate autolysosomes. Bar = 1 μm (F). Quantification of autolysosomes. *n* = 6 IFMs for every group were analyzed (G). Unpaired Student's *t*‐test. *p < 0.05. Data are from at least three biological replicates and presented as mean ± SEM. (H–J) Immunoblotting detection of soluble and insoluble ubiquitinated proteins in young (5‐day‐old), middle (15‐day‐old), and old (30‐day‐old) thoraces of wildtype flies (Mhc‐Gal4>*w* ^*1118*^) (H). Quantitation of soluble (I) and insoluble (J) ubiquitinated proteins/β‐actin. One‐way ANOVA followed by Tukey's test. **p < 0.01, *p < 0.05, n.s., no significance. Data are from at least three biological replicates and presented as mean ± SEM. (K, L) Mitochondria superoxide radicals staining of IFM from young (5‐day‐old), middle (15‐day‐old) and old (30‐day‐old) wildtype flies (Mhc‐Gal4>*w* ^*1118*^). Samples were stained with MitoSOX (red) (K). Bar = 10 μm. Quantification of free superoxide radicals. *n* = 6 IFMs for each group (L). One‐way ANOVA followed by Tukey's test. ****p < 0.0001, ***p < 0.001. Data are from at least three biological replicates and presented as mean ± SEM. (M) ATP contents of thoraces from young (5‐day‐old) and old (30‐day‐old) wildtype flies (Mhc‐Gal4>*w* ^*1118*^). Results were against the protein levels. Unpaired Student's *t*‐test. **p < 0.01. Data are from at least three biological replicates and presented as mean ± SEM. (N) Climbing index of young (5‐day‐old) and old (30‐day‐old) wildtype flies (Mhc‐Gal4>*w* ^*1118*^). *n* > 100, Unpaired Student's *t*‐test. ****p < 0.0001. Data are from at least three biological replicates and presented as mean ± SEM.

We next analyzed mitophagy in IFMs of *Drosophila* during the aging process using the mito‐SRAI sensor. Compared with 5‐day‐old flies, mitophagy was significantly reduced by approximately 37% in 15‐day‐old flies and by 70% in 30‐day‐old flies (Figure 1A,B). Immunoblotting analysis revealed an age‐dependent accumulation of lipidated Atg8a‐II (Figure 1C), indicative of impaired autophagic flux. Consistently, levels of Refractory to Sigma P [Ref(2)P], the sole *Drosophila* orthologue of p62, were significantly elevated in muscles of 15‐ and 30‐day‐old flies compared to that of 5‐day‐old flies (Figure 1D,E). Transmission electron microscopy (TEM) further showed age‐associated increases in mitochondrial electron density and cristae abnormalities (Figure 1F) accompanied by a significant reduction of autolysosomes (Figure 1F,G). Furthermore, detergent‐insoluble ubiquitinated proteins were accumulated in thoracic tissues with age (Figure 1H–J). Elevated levels of mitochondrial ROS and declined ATP production were detected in aged flies (30‐day‐old) (Figure 1K–M). Functionally, locomotor capacity of old flies (30‐day‐old) was reduced to 37.5% of that observed in their young control flies (5‐day‐old) (Figure 1N). These findings demonstrate an aging‐dependent decline in mitophagy and mitochondrial metabolism in *Drosophila* IFMs.

### 2.3 Expression of BNIP3 in Muscles Prolongs Lifespan of *Drosophila* by Regulating Mitophagy

**FIGURE 2:**
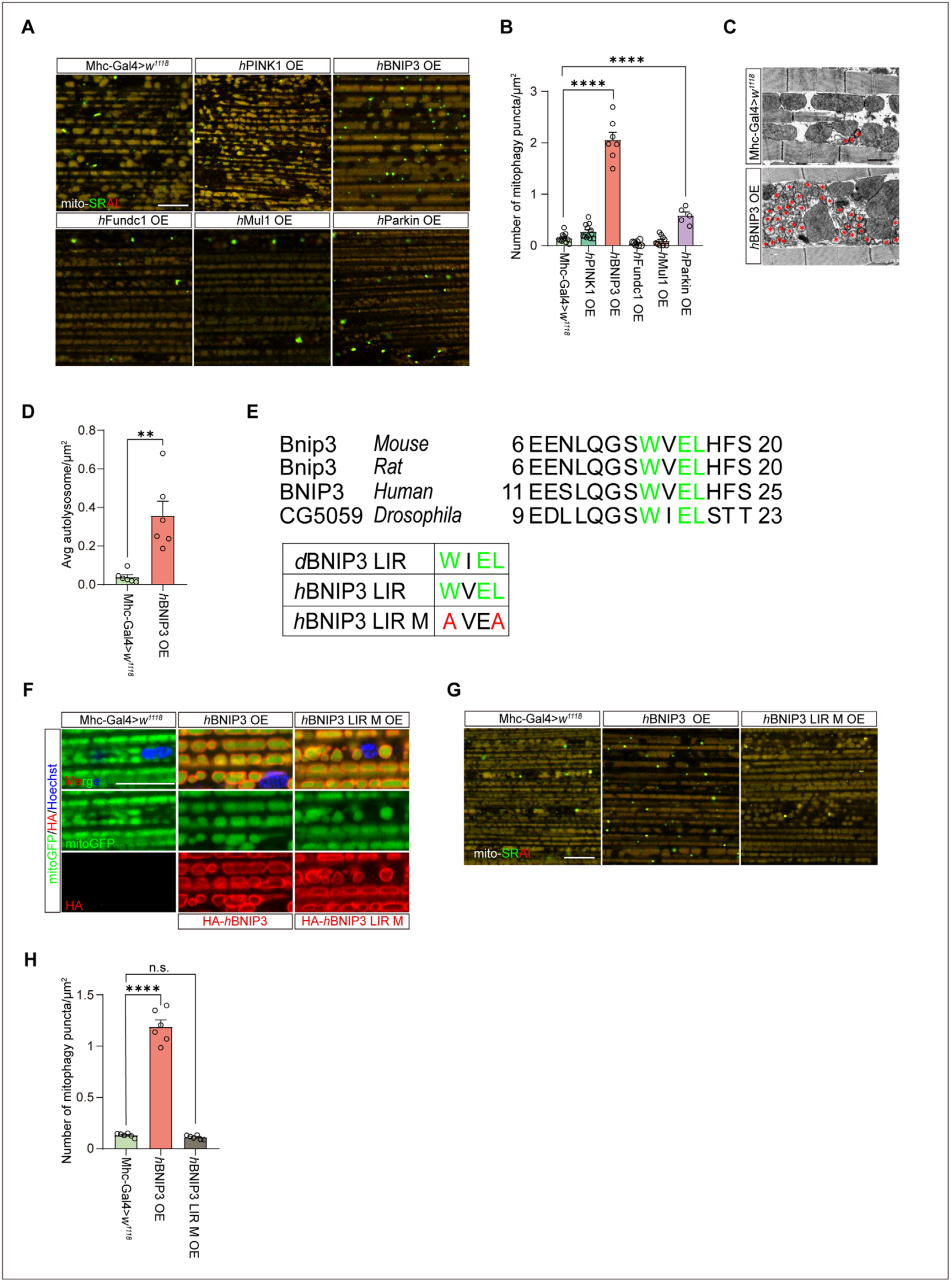
Induction of BNIP3 significantly enhances mitophagy in *Drosophila* IFMs. (A, B) Mito‐SRAI analysis in IFMs for young (5‐day‐old) flies. Young wildtype flies (Mhc‐Gal4>*w* ^*1118*^) and young flies overexpressing *h*PINK1 (*h*PINK1 OE), *h*BNIP3 (*h*BNIP3 OE), *h*Fundc1 (*h*Fundc1 OE), hMul1 (*h*Mul1 OE) or *h*Parkin (*h*Parkin OE) were analyzed. Bar = 10 μm (A). Quantification of mito‐SRAI labeled mitophagy. *n* = 12 (Mhc‐Gal4>*w* ^*1118*^), 12 (*h*PINK1 OE), 7 (*h*BNIP3 OE), 7 (*h*Fundc1 OE), 10 (*h*Mul1 OE) and 5 (*h*Parkin OE) IFMs (B). One‐way ANOVA followed by Tukey's test. ****p < 0.0001. Data are from at least three biological replicates and presented as mean ± SEM. (C, D) TEM images of IFMs from young (5‐day‐old) wildtype flies (Mhc‐Gal4>*w* ^*1118*^) and young flies overexpressing hBNIP3 (*h*BNIP3 OE). Red asterisks label autolysosomes. Bar = 1 μm (C). Quantification of autolysosomes. *n* = 6 IFMs for every group (D). Unpaired Student's *t*‐test. **p < 0.01. Data are from at least three biological replicates and presented as mean ± SEM. (E) Protein sequence alignment of LIR motif from mouse, rat, human BNIP3 and fly (CG5059). Three essential amino acids are indicated in green. Mutation for human BNIP3 LIR motif was indicated in red. (F) Immunofluorescent detection of BNIP3 in mitochondria of IFMs in young (5‐day‐old) flies. BNIP3 localization in IFM from control flies (Mhc‐Gal4>*w* ^*1118*^) and flies overexpressing either *h*BNIP3 (*h*BNIP3 OE) or LIR mutated *h*BNIP3 (*h*BNIP3 LIR M OE) was analyzed (red). mitoGFP (green) and nuclei (blue) were also detected. Bar = 5 μm. (G, H) Mito‐SRAI detection in IFM expressing *h*BNIP3 variants. Young (5‐day‐old) wildtype flies (Mhc‐Gal4>*w* ^*1118*^) and young flies overexpressing *h*BNIP3 (*h*BNIP3 OE) and *h*BNIP3 LIR mutant (*h*BNIP3 LIR M OE). Bar = 10 μm (G). Quantitation of mito‐SRAI labeled mitophagy. *n* = 6 IFMs for each group (H). One‐way ANOVA followed by Tukey's test. ****p < 0.0001, n.s. no significance. Data are from at least three biological replicates and presented as mean ± SEM.

To further investigate the link between mitophagy and aging, we examined the effects of five mitophagy‐associated genes, including *PINK1* (Geisler et al. 2010), *BNIP3* (Zhang et al. 2016), *Fundc1* (Xu et al. 2023), *Mul1* (Puri et al. 2019), and *Parkin* (Geisler et al. 2010), on mitophagy in IFMs. These mitophagy genes are evolutionarily conserved between humans and *Drosophila*, as demonstrated by sequence comparisons and cross‐species functional rescue experiments (Clark et al. 2006; Greene et al. 2003; Park et al. 2006; Xu et al. 2023; Yang et al. 2006; Yun et al. 2014; Zhang et al. 2016). Moreover, their endogenous expression remained largely unchanged during muscle aging (Figure S3A). In the muscle of 5‐day‐old flies, overexpression of human *PINK1* (*hPINK1*) and *Parkin* (*hParkin*) moderately but significantly enhanced mitophagy, whereas overexpression of human *Fundc1* (*hFundc1*) and *Mul1* (*hMul1*) had little effect compared with matched controls. In contrast, overexpression of human *BNIP3* (*hBNIP3*) in the muscle of 5‐day‐old flies led to a 13‐fold increase in mitophagy, as detected by mito‐SRAI (Figure 2A,B; Figure S3B–F). Consistently, TEM analysis revealed a significant increase in autolysosomes in the IFMs of flies overexpressing *hBNIP3* compared with controls (Figure 2C,D). BNIP3 regulates mitophagy by interacting with Atg8a through its LC3‐interacting‐region (LIR) motifs (Hanna et al. 2012). The BNIP3 LIR motif is highly conserved among *Drosophila*, mice, and humans (Figure 2E). While both exogenous wild‐type (WT) and LIR‐mutant BNIP3 were properly localized to the outer mitochondrial membrane in IFMs, LIR‐mutant BNIP3 failed to enhance mitophagy despite normal mitochondrial targeting (Figure 2F–H). Thus, the LIR domain of BNIP3 is indispensable for its mitophagic activity in flies, indicating that BNIP3 regulates mitophagy by directly interacting with Atg8a via its LIR domain.

**FIGURE 3:**
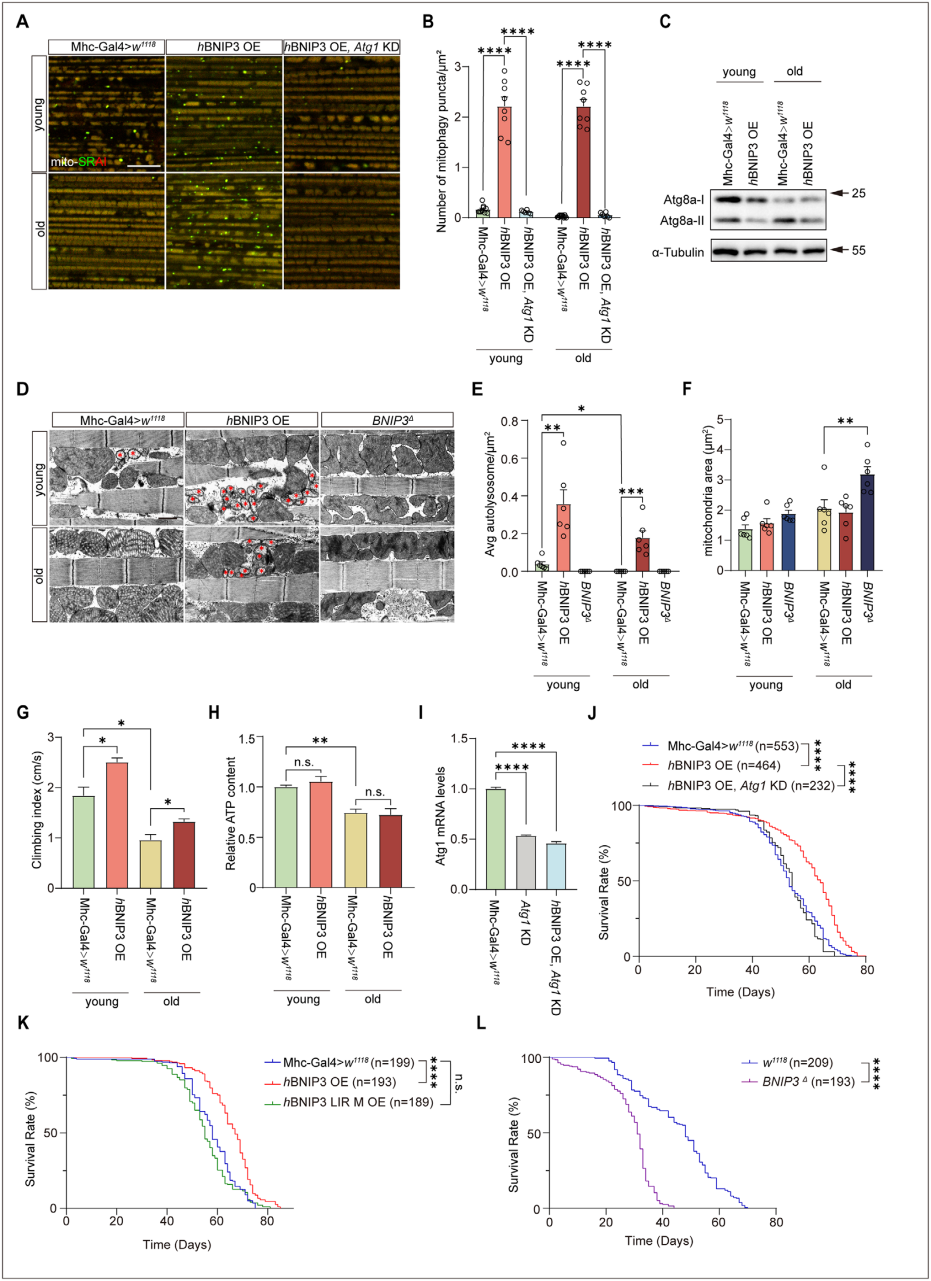
BNIP3 is important to maintain healthy lifespan through regulating mitophagy in *Drosophila* muscles. (A, B) Mito‐SRAI detection in IFM of aging flies. Young (5‐day‐old) and old (30‐day‐old) flies, including wildtype flies (Mhc‐Gal4>*w* ^*1118*^), flies overexpressing *h*BNIP3 (*h*BNIP3 OE), and flies overexpressing *h*BNIP3 together with Atg1 RNAi (*h*BNIP3 OE, *Atg1* KD) were analyzed. Bar = 10 μm (A). Quantification of mito‐SRAI labeled mitophagy. *n* = 10 (Mhc‐Gal4>*w* ^*1118*^, young), 8 (*h*BNIP3 OE, young), 6 (*h*BNIP3 OE, *Atg1* KD, young), 13 (Mhc‐Gal4>*w* ^*1118*^, old), 8 (*h*BNIP3 OE, old) and 6 (*h*BNIP3 OE, Atg1 KD, old) IFMs (B). Two‐way ANOVA followed by Tukey's test. ****p < 0.0001. Data are from at least three biological replicates and presented as mean ± SEM. (C) Immunoblotting analysis of ATG8a in aging flies. Thoraces from young (5‐day‐old) and old (30‐day‐old) flies, including wildtype flies (Mhc‐Gal4>*w* ^*1118*^) and flies overexpressing *h*BNIP3 (*h*BNIP3 OE), were analyzed. Note that overexpression of *h*BNIP3 increased cleavage of ATG8a accompanied by reduced ATG8a‐II accumulation compared to control in old flies. (D–F) TEM images of mitochondria in IFMs. Morphology of mitochondria was imaged in IFMs derived from young (5‐day‐old) and old (30‐day‐old) flies, including wildtype flies (Mhc‐Gal4>*w* ^*1118*^) and flies overexpressing *h*BNIP3 (*h*BNIP3 OE) and flies harboring inactive mutant *BNIP3* ^*Δ*^. Red asterisks indicate autolysosomes. Bar = 1 μm (D). Quantification of autolysosomes. *n* = 6 IFMs for each group (E). Quantification of mitochondria area. *n* = 6 IFMs for each group (F). Two‐way ANOVA followed by Tukey's test. ***p < 0.001, **p < 0.01, *p < 0.05. Data are from at least three biological replicates and presented as mean ± SEM. (G) Climbing assay of aging flies. Climbing assay results of young (5‐day‐old) and old (30‐day‐old) flies, including wildtype flies (Mhc‐Gal4>*w* ^*1118*^) and flies overexpressing *h*BNIP3 (*h*BNIP3 OE). Two‐way ANOVA followed by Tukey's test. *p < 0.05. Data are from at least three biological replicates and presented as mean ± SEM. (H) ATP content analysis. Thoraces derived from young (5‐day‐old) and old (30‐day‐old) wildtype flies (Mhc‐Gal4>*w* ^*1118*^) and flies overexpressing *h*BNIP3 (*h*BNIP3 OE) were measured and normalized against the protein levels. Two‐way ANOVA followed by Tukey's test. **p < 0.01, n.s., no significance. Data are from at least three biological replicates and presented as mean ± SEM. (I) Verification of *Atg1* RNAi in muscle tissue of young (5‐day‐old) flies. Thoraces from wildtype control flies (Mhc‐Gal4>*w* ^*1118*^), flies with muscle‐specific *Atg1* RNAi (*Atg1* KD), and flies co‐expressing *h*BNIP3 and *Atg1* RNAi (*h*BNIP3 OE, *Atg1* KD) were analyzed. One‐way ANOVA followed by Tukey's test. ****p<0.0001. Data are from at least three biological replicates and presented as mean ± SEM. (J) The lifespan analysis. Wildtype flies (Mhc‐Gal4>*w* ^*1118*^), flies overexpressing *h*BNIP3 (*h*BNIP3 OE), and flies overexpressing *h*BNIP3 combined with Atg1 RNAi (*h*BNIP3 OE, *Atg1* KD) were assayed for lifespan. n number is indicated in the figure. Log‐rank test. ****p < 0.0001. (K) The lifespan analysis. Wildtype flies (Mhc‐Gal4>*w* ^*1118*^) and flies overexpressing either *h*BNIP3 (*h*BNIP3 OE) or *h*BNIP3 LIR mutant (*h*BNIP3 LIR M OE) were assayed for lifespan. The n number is indicated in the figure. Log‐rank test. ****p < 0.0001, n.s., no significance. (L) The lifespan analysis. Wildtype flies (*w* ^*1118*^) and *BNIP3* ^Δ^ flies were assayed for lifespan. *n* number is indicated in the figure. Log‐rank test. ****p < 0.0001.

BNIP3 regulates mitophagy and mitochondrial function to contribute to mitigation of muscle aging in mammals (Irazoki et al. 2022). We next investigated whether BNIP3 could alleviate aging of *Drosophila*. Muscle‐specific induction of *BNIP3* significantly increased mitophagy in aged flies (30‐day‐old) compared with young controls (5‐day‐old) (Figure 3A,B). This was further verified by immunoblotting analysis of Atg8a (Figure 3C). Notably, *Atg1* knockdown suppressed BNIP3‐induced mitophagy (Figure 3A,B), indicating that BNIP3 rescues mitophagy during aging through an Atg1‐dependent mechanism. Overexpression of *BNIP3* in IFMs substantially restored autolysosome abundance, preserved mitochondrial cristae integrity, and improved climbing ability (Figure 3D–G). In contrast, the *BNIP3* deletion mutant *BNIP3* ^*Δ*^ (Wang et al. 2023) caused mitochondrial morphological abnormality in both young and old flies (Figure 3D–F). The decreased number of autolysosomes in both young and old *BNIP3* ^*Δ*^ flies, along with the enlarged mitochondria area in old *BNIP3* ^*Δ*^ flies (Figure 3E,F), was highly consistent with TEM phenotypes during the pupal stage (Taoka et al. 2025). Similar phenotypes were also observed in aged muscle‐specific *BNIP3* KD flies (Figure S3G–J). Together, these results suggest that BNIP3 is essential for mitophagy. However, BNIP3 did not rescue the age‐induced decline in ATP levels (Figure 3H). It is possible that aged mitochondria are less functional in ATP production but are not rescued by mitophagy. Remarkably, muscle‐specific expression of BNIP3 significantly extended the lifespan of flies compared with controls (Figure 3I; Figure S4A–D; Table S1). This lifespan extension was partially abolished by *Atg1* knockdown (Figure 3I,J). Given the efficiency of *Atg1* knockdown (Figure 3I) and the observation that *h*BNIP3 OE, *Atg1* KD flies did not show mitophagy levels below those of the control group in either young or old flies (Figure 3B), it is possible that residual Atg1 expression remained sufficient to support autophagic function and thereby prevent a further decline in mitophagy. Moreover, expression of the BNIP3 LIR mutation in muscles did not extend lifespan (Figure 3K). In contrast, *BNIP3* ^*Δ*^ shortened lifespan (Figure 3L), whereas muscle‐specific *BNIP3* KD did not produce the same phenotype, likely attributable to insufficient knockdown efficiency (Figure S3K). Hence, expression of *BNIP3* in muscle tissues prolongs *Drosophila* lifespan through a mitophagy‐dependent mechanism.

### 2.4 BNIP3 Inhibits Age‐Dependent AMP Expression in IFMs

Transcriptomic profiling revealed that muscle tissues of aged flies (Mhc‐Gal4>^*w1118*^, 30‐day‐old) showed significant upregulation of Relish target AMPs, including four *Attacins* (*ATT*), four *Cecropins* (*Cec*), one *Defensin* (*Def*), and two *Diptericins* (*Dpt*) (Figure S5A,B,E). Muscle‐specific expression of *BNIP3* markedly suppressed the age‐induced AMPs upregulation (Figure S5C–E). The increased expression of Relish target genes *AttA*, *CecA1*, and *CecC* in aged flies was further verified by real‐time qPCR (Figure S5F–H). Consistently, the expression of *AttA*, *CecA1*, and *CecC* was further upregulated in aged *BNIP3* ^*Δ*^ flies and aged muscle‐specific *BNIP3* RNAi flies (30‐day‐old) (Figure S6A–F). Moreover, *Atg1* knockdown resulted in increased expression of Relish target genes in aged flies expressing *BNIP3* (Figure S6G–I). Thus, BNIP3 in muscle tissues suppresses the Relish signaling, which is dependent, at least in part, on mitophagy. To exclude microbial influences on Relish target gene activation, flies were reared on antibiotic‐enriched food (Figure S7A). Results showed that Relish target genes were upregulated with aging while this upregulation was inhibited by expressing BNIP3 (Figure S7B–D). Meanwhile, BNIP3 expression in muscle extended the lifespan of flies maintained on antibiotic food (Figure S7E). Together, results suggest that *BNIP3* promotes muscle homeostasis and longevity by suppressing expression of Relish target AMPs via a mitophagy‐mediated mechanism in *Drosophila*.

### 2.5 Relish Signaling Activation in *Drosophila* Muscle Regulates Longevity

**FIGURE 4:**
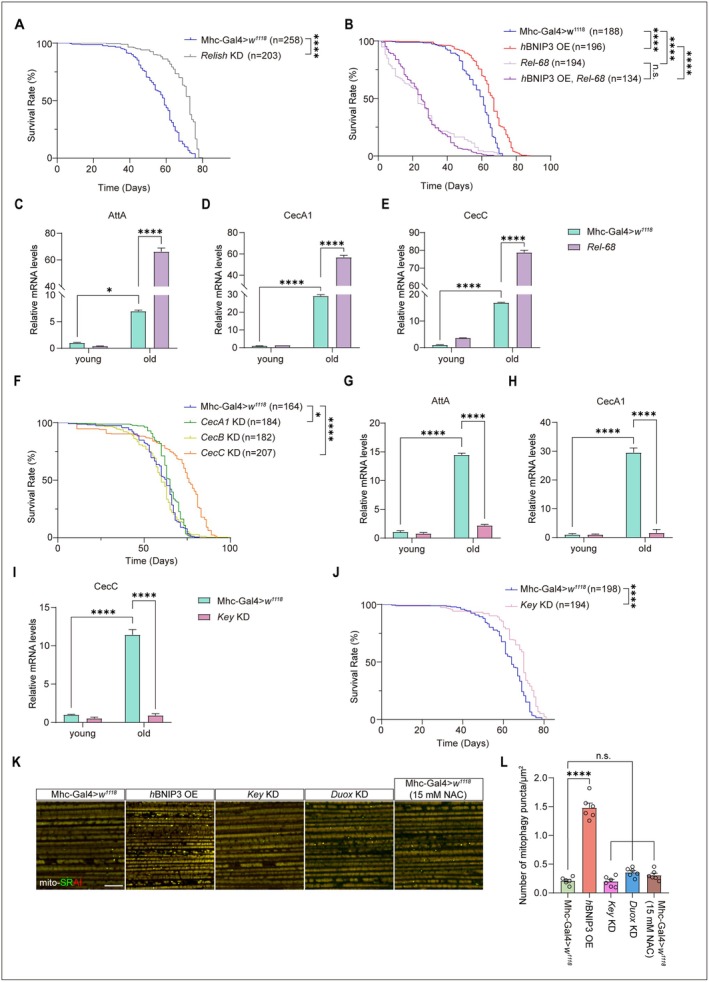
Muscle‐derived Relish activation regulates longevity in *Drosophila*. (A) The lifespan analysis of *Relish* KD flies. Wildtype flies (Mhc‐Gal4>*w* ^*1118*^) and flies with Relish knockdown (*Relish* KD) were assayed for lifespan. n number is indicated in the figure. Log‐rank test. ****p < 0.0001. (B) The lifespan analysis was performed for wildtype flies (Mhc‐Gal4>*w* ^*1118*^) and flies overexpressing either *h*BNIP3 (*h*BNIP3 OE), *Rel‐68*, or both *h*BNIP3 and *Rel‐68 (h*BNIP3 OE, *Rel‐68*). *n* number is indicated in the figure. Log‐rank test. ****p < 0.0001, n.s., no significance. (C–E) Quantitative analysis of AttA, CecA1 and CecC by qPCR. Thoraces derived from young (5‐day‐old) and old (30‐day‐old) flies, including wildtype flies (Mhc‐Gal4>*w* ^*1118*^) and flies expressing *Rel‐68* were analyzed. Two‐way ANOVA followed by Tukey's test. ****p<0.0001, *p<0.05. Data are from at least three biological replicates and presented as mean ± SEM. (F) The lifespan analysis of *CecA1* KD, *CecB* KD, and *CecC* KD flies. Wildtype flies (Mhc‐Gal4>*w* ^*1118*^) and flies with CecA1 knockdown (*CecA1* KD), CecB knockdown (*CecB* KD), or CecC knockdown (*CecC* KD) were assayed for lifespan. *n* number is indicated in the figure. Log‐rank test. ****p<0.0001, *p<0.05. (G–I) Expression of AttA, CecA1, and CecC. Thoraces derived from young (5‐day‐old) and old (30‐day‐old) flies, including wildtype flies (Mhc‐Gal4>*w* ^*1118*^) and flies with *Key* knockdown (*Key* KD), were analyzed. Two‐way ANOVA followed by Tukey's test. ****p < 0.0001. Data are from at least three biological replicates and presented as mean ± SEM. (J) The lifespan analysis of *Key* KD flies. Wildtype flies (Mhc‐Gal4>*w* ^*1118*^) and flies with *Key* knockdown (*Key* KD) were assayed for lifespan. n number is indicated in the figure. Log‐rank test. ****p < 0.0001. (K, L) Mitophagy in IFMs of flies. Mitophagy in IFMs derived from wildtype flies (Mhc‐Gal4>*w* ^*1118*^), flies expressing either *h*BNIP3 (*h*BNIP3 OE), *Key* RNAi (*Key* KD), or *Duox* RNAi (*Duox* KD) and wildtype flies treated with 15 mM NAC treatment (Mhc‐Gal4>*w* ^*1118*^, 15 mM NAC) were detected with mito‐SRAI. Bar = 10 μm (K). Quantification of mitophagy detected by mito‐SRAI. *n* = 6 IFMs for each group (L). One‐way ANOVA followed by Tukey's test. ****p < 0.0001, n.s., no significance. Data are from at least three biological replicates and presented as mean ± SEM.

Age‐associated AMPs upregulation, a hallmark of inflammatory aging, has been primarily studied in gut, glia, and neurons (Kounatidis et al. 2017; Landis et al. 2004; Zerofsky et al. 2005). Muscle‐specific Relish RNAi in flies resulted in a significant extension of lifespan compared with control flies (Figure 4A). Conversely, overexpression of an active, cleaved form of Relish (*Rel‐68*) in muscles markedly shortened lifespan (Figure 4B) and significantly induced AMPs expression in aged flies (Figure 4C–E). Importantly, overexpression of *BNIP3* failed to rescue lifespan reduction caused by Relish activation in *Drosophila* (Figure 4B). Therefore, BNIP3 functions upstream of aging‐induced Relish activation. Together, Relish activation in muscles shortens *Drosophila* lifespan via increasing expression of AMPs. Muscle‐specific knockdown of either *CecC* or *CecA1* significantly increased lifespan of *Drosophila* compared with the control flies (Figure 4F). Notably, *CecC* knockdown produced a greater lifespan extension of flies than *CecA1* knockdown (Figure 4F). In contrast, knockdown of *CecB*, *DptA*, *AttA*, or *AttC* had little effect on the lifespan of flies compared with control flies (Figure S8A,B). Results suggest that *Cecropins*, particularly *CecA1* and *CecC*, accelerate systemic aging in *Drosophila* through Relish‐mediated inflammatory signaling.

We next dissected the Relish pathway to determine the molecular mechanism of muscle‐mediated aging (S8C‐E). The Relish pathway is initiated upon recognition of bacterial peptidoglycan (PGN) by either PGRP‐SC1b in the hemolymph or PGRP‐LE in cytoplasm (Kleino et al. 2017) (Figure 4G). Muscle‐specific PGRP‐SC1b RNAi had no effect on *Drosophila* lifespan (Figure S8C). Together with our findings under germ‐free conditions (S7B‐D), results suggest that BNIP3 regulates Relish signaling in muscle independently of the external microbial environment, likely through an intracellular autoregulatory response. In this context, mitochondrial DNA (mtDNA) is known to activate NF‐κB via the cGAS/STING signaling pathway (Hoffmann 2003; Jiménez‐Loygorri et al. 2024; Martin et al. 2018). Quantitative PCR analysis revealed significantly higher levels of mitochondrial DNA (mtDNA) release in aged fly muscles (30‐day‐old) compared with young muscles (5‐day‐old). This age‐dependent mtDNA release was significantly suppressed by *BNIP3* expression (Figure S9A–C). However, RNAi‐mediated inhibition of either the cGAS/STING (Figure S9D–F) or Eya (Fedele et al. 2022) pathways (Figure S9G–I) failed to suppress expression of *AttA*, *CecA1*, or *CecC* in aged flies, suggesting aging‐related activation of the Relish signaling was independent of STING signaling. Furthermore, Relish activation is also known to depend on Tak1/Tab2 complex and IKK complexes (Hoffmann 2003). Notably, muscle‐specific knockdown of *Key* (the γ subunit of the IKK complex) suppressed age‐induced AMPs gene expression in IFMs (Figure 4G–I) and significantly extended lifespan of flies (Figure 4J, S8D). In contrast to *BNIP3* overexpression, *Key* knockdown in muscles did not significantly affect mitophagy (Figure 4K,L). This result may indirectly indicate that Key acts downstream of *BNIP3*‐mediated mitophagy. Together, aging‐induced Relish activation in fly muscle is mediated by *Key* via a mitochondria‐dependent mechanism.

### 2.6 ROS‐Regulated Relish Activation Links Mitochondrial Stress to Inflammaging in *Drosophila* Muscles

**FIGURE 5:**
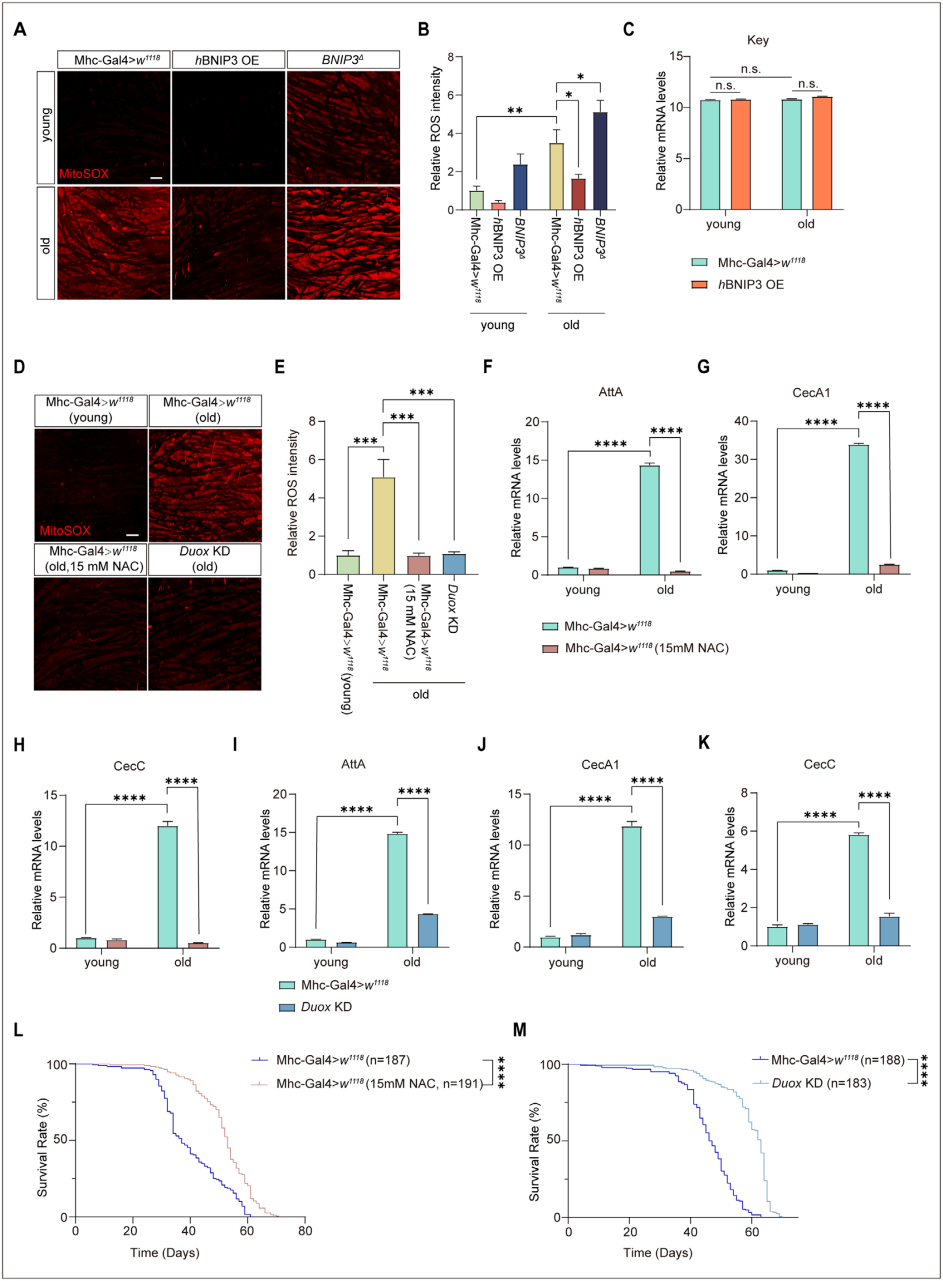
ROS inhibition increases lifespan and suppresses aging‐dependent Relish signaling. (A, B) *BNIP3* ^Δ^ increases mitochondrial ROS detection. IFMs derived from young (5‐day‐old) and old (30‐day‐old) flies, including wildtype flies (Mhc‐Gal4>*w* ^*1118*^), flies overexpressing *h*BNIP3 (*h*BNIP3 OE), and *BNIP3* ^Δ^ mutant flies were stained with MitoSOX. Mitochondria superoxide radicals were shown as red. Bar = 10 μm (A). Quantification of ROS in IFMs. *n* = 8 (Mhc‐Gal4>*w* ^*1118*^, young), 7 (*h*BNIP3 OE, young), 4 (*BNIP3* ^*Δ*^, young), 10 (Mhc‐Gal4>*w* ^*1118*^, old), 10 (*h*BNIP3 OE, old) and 6 (*BNIP3* ^*Δ*^, old) IFMs were analyzed (B). Two‐way ANOVA followed by Tukey's test. **p < 0.01, *p < 0.05. Data are from at least three biological replicates and presented as mean ± SEM. (C) Key Expression. Data is from *Drosophila* thoraces RNA‐seq. Young (5‐day‐old) and old (30‐day‐old) flies, including wildtype flies (Mhc‐Gal4>*w* ^*1118*^) and flies overexpressing *h*BNIP3 expression (*h*BNIP3 OE) were analyzed. Two‐way ANOVA followed by Tukey's test. n.s., no significance. Data are presented as mean ± SEM. (D, E) Aging induced ROS production is inhibited by *Duox* KD. IFMs derived from young (5‐day‐old) and old (30‐day‐old) flies, including wildtype flies (Mhc‐Gal4>*w* ^*1118*^) and flies with *Duox* knockdown (*Duox* KD) were stained with MitoSOX. Mitochondrial ROS is shown as Red. IFMs of control flies treated with 15 mM NAC were included as a positive control (lower left panel). Bar = 10 μm (D). Quantification of mitochondrial ROS is shown. *n* = 9 (Mhc‐Gal4>*w* ^*1118*^, young), 9 (Mhc‐Gal4>*w* ^*1118*^, old), 6 (Mhc‐Gal4>*w* ^*1118*^, old, 15 mM NAC) and 7 (*Duox* KD, old) (E). Two‐way ANOVA followed by Tukey's test. ***p < 0.001. Data are from at least three biological replicates and presented as mean ± SEM. (F–H) NAC treatment inhibits aging‐induced expression of AttA, CecA1, and CecC. Thoraces derived from young (5‐day‐old) and old (30‐day‐old) wildtype flies (Mhc‐Gal4>*w* ^*1118*^) with or without 15 mM NAC treatment are qPCR analyzed. Two‐way ANOVA followed by Tukey's test. ****p < 0.0001. Data are from at least three biological replicates and presented as mean ± SEM. (I–K) *Duox* KD inhibits aging‐induced expression of AttA, CecA1, and CecC. Thoraces derived from young (5‐day‐old) and old (30‐day‐old) wildtype flies (Mhc‐Gal4>*w* ^*1118*^) and flies with *Duox* knockdown (*Duox* KD) were analyzed with qPCR. Two‐way ANOVA followed by Tukey's test. ****p < 0.0001. Data are from at least three biological replicates and presented as mean ± SEM. (L) The lifespan extension by NAC treatment. Wildtype flies (Mhc‐Gal4>*w* ^*1118*^) treated with or without 15 mM NAC were assayed for lifespan. *n* number is indicated in the figure. Log‐rank test. ****p < 0.0001. (M) *Duox* KD increases lifespan of flies. Wildtype control flies (Mhc‐Gal4>*w* ^*1118*^) and flies with *Duox RNAi* (*Duox* KD) were assayed for lifespan. *n* number is indicated in the figure. Log‐rank test. ****p < 0.0001.

ROS production in the gut has been shown to regulate lifespan in *Drosophila* (Iatsenko et al. 2018) and to activate NF‐κB signaling in mammals (Morgan and Liu 2011). Consistently, ROS levels were significantly elevated in muscles of aged flies (30‐day‐old) compared with young control flies (5‐day‐old), and this increase was effectively suppressed by BNIP3 (Figure 5A,B). By contrast, *BNIP3* ^*Δ*^ increased ROS production in both young (5‐day‐old) and aged flies (30‐day‐old) (Figure 5A,B). RNA‐seq analysis revealed that *BNIP3* overexpression in muscle did not alter transcriptional expression of *Key* (Figure 5C). Since ROS promotes NF‐κB activation in mammalian cells, these data suggest that ROS may regulate Relish activation in *Drosophila* muscle by modulating Key function. N‐acetyl‐L‐cysteine (NAC), often used as an antioxidant, scavenges ROS directly or by promoting the synthesis of glutathione (GSH) (Schmitt et al. 2015). NADPH dual oxidases (Duox) are responsible for producing hydrogen peroxide, a ROS, through utilizing NADPH as an electron donor and molecular oxygen (Chakrabarti and Visweswariah 2020; Zhai et al. 2018). Increased ROS in aging *Drosophila* muscle was effectively inhibited by either NAC treatment or *Duox* knockdown (Figure 5D,E). Furthermore, NAC treatment and *Duox* knockdown markedly suppressed the age‐associated increase in AMP expression, including AttA, CecA1, and CecC (Figure 5F–K). Notably, both NAC and muscle‐specific knockdown of *Duox* significantly increased lifespan of *Drosophila* (Figure 5L,M). Results suggest that ROS production in muscle contributes to reduced lifespan of *Drosophila* via a Relish signaling‐mediated mechanism. Interestingly, neither NAC treatment nor *Duox* knockdown in muscle increases mitophagy (Figure 4K,L), suggesting that BNIP3‐mediated mitophagy acts primarily through ROS elimination to maintain muscle and organismal homeostasis.

Given that *BNIP3* ^*Δ*^ shortened lifespan (Figure 3L), activation of Relish in aged flies (Figure S6A–C), and accumulation of ROS in both young (5‐day‐old) and aged (30‐day‐old) flies (Figure 5A,B), we performed rescue experiments in *BNIP3* ^*Δ*^ flies to better align these results with the conclusions drawn from BNIP3 overexpression. NAC treatment suppressed the increase in ROS production in *BNIP3* ^*Δ*^ flies at both ages (Figure S10A,B). Moreover, NAC rescued the shortened lifespan of *BNIP3* ^*Δ*^ flies and attenuated Relish activation in aged *BNIP3* ^*Δ*^ flies (Figure S10C–F). Notably, muscle‐specific knockdown of either *Key* or *Duox* suppressed Relish activation in *BNIP3* RNAi flies (Figure S10G–I). These results support the conclusion that BNIP3‐dependent mitophagy preserves muscle homeostasis by promoting ROS clearance.

### 2.7 *BNIP3* Expression in Muscles Alleviates Aging‐Related Neurodegeneration in *Drosophila*

**FIGURE 6:**
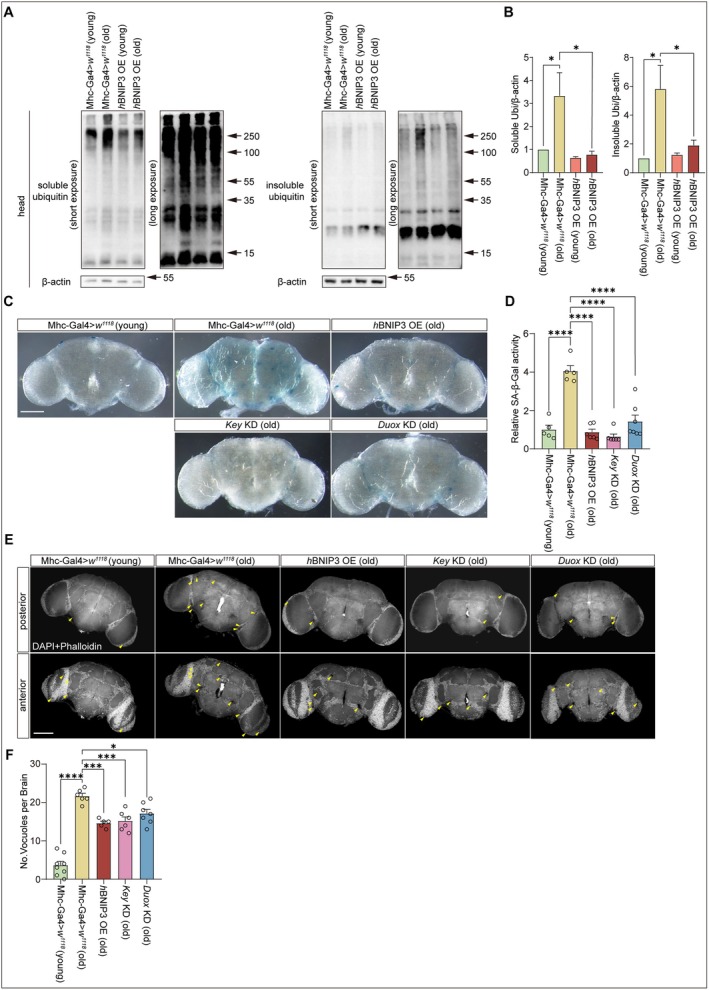
Expression of BNIP3 in skeletal muscles suppresses aging‐dependent neurodegeneration in *Drosophila*. (A, B) BNIP3 suppresses aging‐induced insoluble protein accumulation. Ubiquitinated Triton X‐100 soluble and insoluble proteins in fly heads are detected by immunoblotting analysis with an anti‐ubiquitin antibody. Wildtype flies (Mhc‐Gal4>*w* ^*1118*^) and flies overexpressing *h*BNIP3 (*h*BNIP3 OE) were analyzed. Young, 5‐day‐old; old, 30‐day‐old (A). Quantitation of soluble and insoluble ubiquitinated proteins. Results were normalized with β‐actin (B). Two‐way ANOVA followed by Tukey's test. *p < 0.05. Data are from three independent experiments and presented as mean ± SEM. (C, D) Expression of *h*BNIP3 and knockdown of *Key* or *Duox* inhibit aging‐induced SA‐β‐Gal activity in fly brains. Brains from control flies (Mhc‐Gal4>*w* ^*1118*^) and flies with muscle‐specific expression of *h*BNIP3 (*h*BNIP3 OE), *Key RNAi (Key KD)*, or *Duox RNAi* (*Duox KD*) were analyzed. Young, 5‐day‐old; old, 30‐day‐old. Bar = 100 μm (C). Quantification of SA‐β‐Gal staining for experiments. *n* = 5 (Mhc‐Gal4>*w* ^*1118*^, young), 5 (Mhc‐Gal4>*w* ^*1118*^, old), 6 (*h*BNIP3 OE, old), 5 (*Key* KD, old) and 6 (*Duox* KD, old) (D). One‐way ANOVA followed by Tukey's test. ****p < 0.0001. Data are from at least three biological replicates and presented as mean ± SEM. (E, F) Expression of *h*BNIP3 and knockdown of *Key* or *Duox* suppress aging‐related vacuole formation in fly brains. Brains from control flies (Mhc‐Gal4>*w* ^*1118*^) and flies with muscle‐specific expression of *h*BNIP3 (*h*BNIP3 OE), *Key RNAi (Key* KD), or *Duox RNAi* (*Duox* KD) were analyzed. Young, 5‐day‐old; old, 30‐day‐old. Yellow arrows indicate pathological degenerative vacuoles. Bar = 100 μm (E). Quantification of vacuoles from experiments shown. *n* = 8 (Mhc‐Gal4>*w* ^*1118*^, young), 6 (Mhc‐Gal4>*w* ^*1118*^, old), 5 (*h*BNIP3 OE, old), 6 (*Key* KD, old) and 7 (*Duox* KD, old) (F). One‐way ANOVA followed by Tukey's test. ****p < 0.0001, ***p < 0.001, n.s., no significance. Data are from at least three biological replicates and presented as mean ± SEM.

Muscle health profoundly impacts brain function and the progression of neurodegenerative diseases (Boyle et al. 2009; Demontis and Perrimon 2010; Rai and Demontis 2016; Severinsen and Pedersen 2020; Voss et al. 2013). We next examined the effects of muscle aging on neurodegeneration in fly brains with muscle‐specific genetic manipulations. Aged fly brains (30‐day‐old) showed increased levels of ubiquitinated proteins in both soluble and insoluble fractions compared with young controls (5‐day‐old) (Figure 6A,B). Muscle‐specific expression of *BNIP3* markedly reduced the accumulation of ubiquitinated proteins in both fractions in fly brains (Figure 6A,B). β‐galactosidase (SA‐β‐Gal) activity (Byrns et al. 2024; Dimri et al. 1995) and pathological degenerative vacuoles (Behnke et al. 2021) were also observed in the aged brains (30‐day‐old) but were significantly suppressed by muscle‐specific expression of BNIP3 (Figure 6C–F). Muscle‐specific knockdown of *Key* or *Duox* alleviated age‐associated increase in SA‐β‐Gal activity and accumulation of pathological degenerative vacuoles in brains of aged flies (Figure 6C–F). Notably, muscle‐specific knockdown of *CecA1* or *CecC* produced similar effects (Figure S11A–D). Collectively, these findings suggest that age‐related neurodegeneration in the fly brain is modulated by muscle homeostasis, and that enhancing BNIP3‐mediated mitophagy or suppression of Key/Duox‐dependent inflammatory signaling in muscle mitigates brain aging and proteotoxic stress.

## 3 Discussion

This study makes two major advances. First, we developed a novel *Drosophila* model for in vivo monitoring of mitophagy using mito‐SRAI. This system enables reliable detection of mitophagy across multiple tissues and offers several advantages over existing reporters. Second, we demonstrate that muscle tissue plays a critical role in regulating brain function and systemic aging through a mitophagy‐dependent mechanism. Our findings offer direct mechanistic evidence that BNIP3‐driven mitophagy in muscle not only preserves local muscle integrity and function but also exerts non‐autonomous effects on systemic aging and neurodegeneration.

Genetically encoded reporters such as mito‐QC and mt‐Keima have substantially advanced in vivo analysis of mitophagy in *Drosophila* (Lee et al. 2018). The mito‐QC system utilizes a tandem GFP‐mCherry construct targeted to mitochondria, where GFP fluorescence is quenched upon lysosomal delivery, leaving a stable mCherry signal as a marker of mitolysosomes (Lee et al. 2018). Although robust and widely used, GFP quenching may not always reflect complete degradation, potentially leading to an underestimation of mitophagy activity (Liu et al. 2021). In contrast, mito‐Keima employs a coral‐derived, pH‐sensitive fluorescent protein that undergoes an excitation shift in acidic compartments (Liu et al. 2021). This ratiometric nature enables a more quantitative assessment and is less prone to photobleaching than mito‐QC. However, mito‐Keima requires dual‐excitation imaging platforms and presents spectral overlap issues that limit compatibility with other fluorescent reporters. The recently developed mito‐SRAI reporter addresses several of these limitations. By incorporating two fluorescent proteins with differential lysosomal stability, one resistant and one rapidly degraded, mito‐SRAI enables dynamic measurement of mitophagy flux rather than static accumulation (Katayama et al. 2020). This makes it particularly valuable for studying aging and chronic diseases, where turnover rates are biologically meaningful insights rather than steady‐state levels. In this study, we validate mito‐SRAI in *Drosophila* and demonstrate its ability to detect mitophagy across multiple tissues, including intestine, fat body, and muscle with improved resolution compared to mito‐QC and mito‐Keima. Notably, mito‐SRAI also captures age‐associated changes in mitophagy. These findings establish mito‐SRAI as a powerful addition to the mitochondrial quality control toolkit, offering a more accurate and dynamic approach to study mitophagy in vivo across development, aging, and disease contexts.

Mitophagy is increasingly recognized as a key mechanism for maintaining muscle integrity and function. Multiple pathways regulate muscle mitophagy, including the canonical PINK1/Parkin pathway (Cornelissen et al. 2018), FUNDC1‐dependent mitophagy (Chen et al. 2016), and BNIP3‐mediated mitophagy (Schmid et al. 2022). BNIP3 promotes mitophagy through its LC3‐interacting region (LIR) motif, which enables the selective recruitment of damaged mitochondria to autophagosomes (Hanna et al. 2012; Taoka et al. 2025). Unlike PINK1/Parkin, BNIP3 expression is strongly induced under stress and aging conditions (Irazoki et al. 2022; Schmid et al. 2022). In this study, BNIP3 upregulation alleviates the aging‐associated decline in mitophagy, preserves mitochondrial cristae and suppresses the age‐induced increase in ROS. Consistent with our findings, a recent study demonstrates that BNIP3 enhances mitochondrial cristae formation and promotes fatty acid oxidation, thereby inhibiting cellular senescence (Yamauchi et al. 2024). Collectively, these findings suggest that BNIP3 protects against aging via a mitophagy‐dependent mechanism. We note, however, that Schmid et al. reported that muscle‐specific exogenous BNIP3 expression shortened lifespan, whereas neuronal BNIP3 overexpression extended lifespan, findings that appear to differ from those reported here (Schmid et al. 2022). One possible explanation is the difference in experimental design. Schmid et al. used RU486‐mediated transgenic induction beginning at day 5, whereas this study employed constitutive expression. Such differences in the timing and mode of BNIP3 expression may impose distinct levels of stress on muscle tissue and thereby contribute to the different phenotypic outcomes. Notably, a separate study found that muscle‐specific downregulation of BNIP3 in aged mice accelerates age‐associated muscle atrophy (Irazoki et al. 2022), further supporting a beneficial role for BNIP3 in muscle homeostasis during aging.

Skeletal muscle also functions as an immune organ in response to infection in flies (Yang and Hultmark 2017; Yang et al. 2015; Zhao and Karpac 2021). In mammals, BNIP3 is shown to attenuate age‐related muscle inflammation (Irazoki et al. 2022). Consistently, BNIP3 regulates the expression of AMPs in aging muscle by suppressing the activation of transcription factor Relish. Relish is a key regulator in the immune deficiency (IMD) pathway in *Drosophila* (Hedengren et al. 1999; Wiklund et al. 2009). One mechanism for Relish inhibition by BNIP3 is that BNIP3‐mediated mitophagy effectively reduces mitochondrial ROS production, which serves as an upstream activator of Relish (Chakrabarti and Visweswariah 2020; Nukala et al. 2023). Key, a core component of the *Drosophila* IkB kinase (IKK) complex, is required upstream for Relish activation (Boutros et al. 2002). Our data indicate that Key is necessary for Relish activation in aged muscle, whereas BNIP3‐mediated mitophagy acts primarily to suppress Relish signaling. Notably, BNIP3 does not appear to directly regulate Key‐dependent signaling, but instead modulates Relish activation indirectly by maintaining mitochondrial homeostasis. One plausible mechanism is that BNIP3‐mediated mitophagy reduces mitochondrial ROS production, thereby limiting ROS‐dependent activation of inflammatory signaling pathways that converge on Relish. However, the precise mechanistic link between mitochondrial quality control and Key activity remains unclear. Together, these findings establish a mechanistic connection among mitochondrial dysfunction, Relish activation, and aging.

There is increasing evidence that abnormalities in peripheral tissues contribute to pathogenesis of neurodegeneration (Pluvinage and Wyss‐Coray 2020; Rai et al. 2021; Sampson et al. 2016). Muscle‐to‐brain signaling is a well‐accepted concept. Physical exercise, a primary activator of skeletal muscle, has long been known to enhance learning, memory, and attention (Cotman and Berchtold 2002). The muscle‐to‐brain axis is a powerful regulator of CNS health and function. Targeted overexpression of Transcription Factor E‐B (TFEB) in skeletal muscle reduces Aβ plaque accumulation in the cortex and hippocampus and rescues behavioral neurocognitive deficits in 5xFAD mice (Taha et al. 2024). The same intervention also protects against age‐associated cognitive decline and reduces degenerative neuropathological hallmarks in the MAPT P301S mouse model of tauopathy (Matthews et al. 2023). In this study, activation of BNIP3‐mediated mitophagy in muscles suppresses systemic aging and age‐related neurodegeneration, alleviating pathological aging phenotypes in the brain, including reduction of ubiquitinated protein accumulation, increased activity of β‐galactosidase, and age‐related vacuolization. This study provides compelling evidence for cross‐talk between peripheral tissues and the central nervous system, highlighting a muscle‐to‐brain signaling axis that modulates neurodegeneration through mitochondrial quality control.

Taken together, this study establishes that BNIP3‐mediated mitophagy in muscle regulates systemic aging via suppressing the Relish pathway. Targeting BNIP3 mitophagy pathways may provide a strategy for therapeutic interventions aimed at improving age‐related and aging‐related neurodegenerative diseases.

## 4 Materials and Methods

### 4.1 Plasmids and *Drosophila* Lines

DNA encoding mito‐SRAI, derived from pcDNA3.1/mito‐SRAI (Katayama et al. 2020), was subcloned into pUAST‐attB vector (Unihuaii, Zhuhai, China) using EcoRI and NotI restriction enzymes. Mito‐Keima, derived from pcDNA3.1/mito‐Keima (Yan et al. 2020), was subcloned into pUAST‐attB vector (Unihuaii, Zhuhai, China) using EcoRI and NotI restriction enzymes. HA‐*h*BNIP3 LIR (LC3 Interaction Region) mutants (18‐WVEL‐21 to 18‐AVEA‐21 change) were generated from a pUAST‐HA‐*h*BNIP3(Zhang et al. 2016) with a QuickChange site‐directed Mutagenesis Kit (200,524, Agelient Technologies, Santa Calara, CA) according to the manufacturer's instructions. *h*Parkin, derived from pcDNA3.1/*h*Parkin (Zhang et al. 2016), was subcloned into pUAST‐attB vector. *h*Mul1 cDNA was PCR‐amplified from the universal human cDNA (637,260, Takara Bio, Japan) and subcloned into the pUAST‐attB. All plasmids were sequence verified and purified using an EndoFree Plasmid Maxi kit (12,362, QIAGEN, Venlo, Germany) following instructions. Sequencing primers are shown in Table S2.

Wildtype fly *w* ^*1118*^ (3605) and flies harboring either Mhc‐Gal4 (55133), Cg‐Gal4 (7011), Elav‐Gal4 (458), Repo‐Gal4 (7015), Bam‐GAL4 (80579), PPL‐GAL4 (58768), UAS‐mitoGFP (8443), UAS‐*hPINK1* (52004), UAS‐*FLAG‐Rel.68* (55777), UAS‐*Atg1* RNAi (TRiP.JF02273), UAS‐*Atg5* RNAi (TRiP.JF02703), UAS‐*Atg7* RNAi (TRiP.JF02787), UAS‐*Atg12* RNAi (TRiP.JF02704), UAS‐*Rel* RNAi (TRiP.HM05154), UAS‐*CecA1* RNAi (TRiP.HMC05728), UAS‐*CecB* RNAi (TRiP.HMJ23515), UAS‐*CecC* RNAi (TRiP.HMC05727), UAS‐*DptA* RNAi (TRiP.HMC06322), UAS‐*Key* RNAi (TRiP.HMC04952), UAS‐*Tak1* RNAi (TRiP.JF01384), UAS‐*Tab2* RNAi (TRiP.JF03353), UAS‐*STING* RNAi (TRiP.JF01138), or UAS‐*Duox* RNAi (TRiP.GL00688) were obtained from the *Bloomington Drosophila Stock Center* (BDSC, Bloomington, IN). UAS‐*BNIP3* RNAi lines (v9847, v107493) were obtained from *Vienna Drosophila Resource Center* (VDRC, Vienna, Austria). UAS‐*AttA* RNAi (TH03175.N), UAS‐*AttC* RNAi (TH03360.N), UAS‐*Eya* RNAi (THU4048), UAS‐*PGRP‐SC1b* RNAi (TH03593.N), and UAS‐*PGRP‐LE* RNAi (TH03230.N) were obtained from *TsingHua Fly Center* (THFC, Beijing, China). UAS‐mitoQC, Myo1a‐Gal4, and *BNIP3* ^*Δ*^ */TM6B* were gifts from Dr. Ruoxi Wang. UAS‐mito‐SRAI, UAS‐mito‐Keima, UAS‐HA‐*h*BNIP3 LIR M, UAS‐*h*Parkin, UAS‐flag‐*h*Mul1, and UAS‐myc‐*h*FUNDC1(Xu et al. 2023) were generated in our laboratory.

All transgenic flies were generated as described previously (Wang et al. 2024; Zhang et al. 2016). Multiple independent fly lines were generated and analyzed (UniHuaii, Zhuhai, China). Briefly, constructs with the attB sequence were injected into flies (y^1^w^67c23^; P(CaryP)attP2 and y^1^w^67c23^; P(CaryP)attP40) to initiate the φC31 integrase‐mediated site‐specific integration. The resulted flies (G0) were crossed with double balancer lines to produce the F1 generation. Fly strains were grown on standard cornmeal medium at 25°C.

Throughout the text, young, middle‐aged, and old fruit flies refer to flies aged 5, 15, and 30 days, respectively.

### 4.2 Chemicals and Antibodies

MitoSOX (M36008), Hoechst (33342), LysoTracker Deep Red (L12492), and Rhodamine Phalloidin (F415) were from Thermo Fisher Scientific (Waltham, MA). N‐Acetyl‐L‐cysteine (NAC, ST1546) was from Beyotime (Shanghai, China). Fluoromount Aqueous Mounting Medium (F4680) was from Sigma (Darmstadt, Germany). Protease inhibitor cocktail (11836170001) was from Roche (Basel, Switzerland). ENLITEN ATP Assay System (FF2000) was from Promega (Madison, WI). Ampicillin (HY‐B0522), Metronidazole (HY‐B0318), and Tetracyclin (HY‐A0107) were from MedChemExpress (New Jersey, USA). Antibodies for Atg8a (13733), ubiquitin (43124), HA‐tag (3724), α‐tubulin (12351), PINK1 (6946), flag (14793), myc (2272), and Senescence β‐Galactosidase Staining Kit (9860) were from Cell Signaling Technology (Boston, MA). Parkin (sc‐32,282) antibody was purchased from Santa Cruz Biotechnology (Dallas, TX, USA). β‐actin (ab8224) and Ref(2)P (ab178440) antibodies were from Abcam (Cambridge, UK).

### 4.3 Immunofluorescence and Confocal Microscopy

Immunofluorescence staining was performed as previously described (Gao et al. 2022; Han et al. 2020; Zhang et al. 2016). Briefly, muscles were dissected and fixed in 4% paraformaldehyde in phosphate buffered saline (PBS). After muscles were washed three times in PBS, samples were permeabilized in PBS containing 0.1% Triton X‐100, blocked with 5% normal goat serum in PBS, followed by incubating with primary and secondary antibodies diluted in 5% normal goat serum in PBS. Slides were mounted with Fluoromount.

Fluorescent samples were routinely examined with a Leica TCS SP8 confocal microscope (Wetzlar, Germany) equipped with a Plan‐Apochromat 20×/0.8 NA, 40×/1.4 NA, and a 63×/1.4 NA oil immersion objective lens.

### 4.4 Mitophagy Reporter Imaging and Quantification

Mitophagy detection by mito‐SRAI, mito‐QC, and mito‐Keima was essentially performed as described (Katayama et al. 2020, 2011; McWilliams et al. 2016). Fluorescence of mito‐SRAI was imaged in two channels using one single excitation wavelength (a 458‐nm diode laser) and two emission ranges (465–495 nm for TOLLES and 515–545 nm for YPet). For fluorescent imaging of mito‐QC, pH‐sensitive GFP was excited by a 488 nm diode laser using a 500–550 nm emission range. mCherry was excited by a 561 nm diode laser using a 570–695 nm emission range. Fluorescence of mito‐Keima was imaged in two channels via two sequential excitations (458 nm for “green” and 561 nm for “red”, respectively) using a 570–695 nm emission range. Laser power was set at the lowest output that would allow clear visualization of the fluorescent signal.

To quantify mitophagy puncta detected by mito‐SRAI, TOLLES and YPet fluorescence signals were optimized to reduce background while maintaining clear visualization of the mitochondrial network and green (TOLLES) puncta. The resulting TOLLES‐only puncta, representing mitophagic events, were quantified per unit area in each tissue sample using ImageJ (Version 1.54, NIH, Bethesda, MD). For mito‐QC analysis, GFP and mCherry signals were also adjusted to minimize background and clear visualization of mitochondrial structures. The remaining mCherry‐only puncta, indicative of mitochondria delivered to lysosomes, were counted using ImageJ. For mito‐Keima assay, fluorescent signals were acquired at 458 nm excitation (green) and 561 nm excitation (red) to distinguish mitochondria in neutral and acidic environments, respectively. Mitophagy puncta were identified with red‐positive signals and quantified as a measure of mitophagic activity.

### 4.5 *Drosophila* Tissue Preparation

For fixed mito‐QC and mito‐SRAI, adult testes, intestines, indirect flight muscles, and fat bodies were dissected in PBS and fixed in 4% formaldehyde for 1 h. Tissues were washed in 0.3% PBST twice and mounted using Fluoromount.

For live mito‐Keima and mito‐SRAI, adult testes, intestines, indirect flight muscles, and fat bodies were dissected in Schneider's *Drosophila* medium (SDM, 21720024, Thermo Fisher Scientific, Waltham, MA). Samples were mounted in SDM immediately followed by analysis using a Leica TCS SP8 confocal microscopy (Wetzlar, Germany).

### 4.6 LysoTracker Deep Red Staining

LysoTracker Deep Red staining was essentially done as previously described (He et al. 2019). Third‐instar larvae were transferred from standard food to 20% sucrose (S0389, Millipore Sigma, Darmstadt, Germany) in water for 4 h prior to dissection (Anding et al. 2018). Control larvae were transferred to standard food until dissection. The fat body was dissected in Schneider's *Drosophila* medium (SDM, 21720024, Thermo Fisher Scientific, Waltham, MA). Tissues were incubated for 15 min with 500 nM LysoTracker Deep Red (L12492, Thermo Fisher Scientific, Waltham, MA) diluted in SDM. Samples were mounted in SDM and immediately visualized with a Leica TCS SP8 confocal microscopy (Wetzlar, Germany).

### 4.7 MitoSOX Staining

As previously described (Wang et al. 2024), flies were anesthetized and hemi‐thoraces were dissected in cold Schneider's *Drosophila* medium (SDM, 21720024, Thermo Fisher Scientific, Waltham, MA). Hemi‐thoraces were then incubated in a staining solution containing 5 μM MitoSOX Red (M36008, Thermo Fisher Scientific, Waltham, MA) in SDM for 12 min at RT. Samples were washed twice with SDM (30 s for each), quickly mounted in SDM and imaged within 10–15 min by a Leica TCS SP8 confocal microscopy (Wetzlar, Germany). Staining intensity was quantified using Image J (Version 1.54, NIH, Bethesda, MD).

### 4.8 Immunoblotting

Immunoblotting was performed as described previously (Xiong et al. 2009). Briefly, samples were homogenized and lysed with SDS sample buffer (63 mM Tris–HCl, 10% glycerol, and 2% SDS) containing a protease and phosphatase inhibitor cocktail (11,836,170,001, Roche, Basel, Switzerland). Tissue lysates were sonicated followed by centrifuging at 14,000*g* for 10 min at 4°C. Supernatants were collected. Protein concentrations were determined using the BCA Protein Assay (A65453, Thermo Fisher Scientific). Proteins (15–20 μg) were separated in an SDS‐PAGE gel and transferred onto PVDF membranes (88,518, Thermo Fisher Scientific). After blocking, membranes were incubated overnight at 4°C with appropriate primary antibodies. The membranes were washed with 0.1% PBST and incubated with secondary antibodies for 1 h. Signals were detected with horseradish peroxidase‐labeled antibodies and captured using the ChemiDoc Imaging System. Densitometric quantification of bands was analyzed using Image J (Version 1.54).

### 4.9 Mitochondrial DNA (mtDNA) Isolation and Quantitative PCR

Mitochondrial fractionation was done as previously described (Bryant et al. 2022; Lai, Wang, et al. 2024; Xiong et al. 2009). About 50 thoraces were dissected from flies and homogenized in 1 mL cold HEPES buffer. The homogenate was subsequently divided into two 1.5 mL tubes and designated as “A” and “B.” Sample A was lysed in 250 μL of SDS lysis buffer and heated to 95°C for 15 min, resulting in the “whole cell extract” (WCE). Sample B was lysed in digitonin lysis buffer (150 mM NaCl, 50 mM HEPES, pH 7.4, and 15 μg/mL digitonin) for 10 min at 4°C with rotating. The samples were centrifuged at 950*g* for 5 min at 4°C. The resultant cytosolic supernatants were carefully transferred into fresh tubes and designated as the “cytosolic fraction” (Cyto). Cytosolic fractions were further centrifuged at 17,000*g* for 5 min at 4°C to pellet remaining cellular debris. The pellets were washed three times with 1 mL of ice‐cold PBS, spinning at 950*g* for 3 min at 4°C. The pellets were resuspended in 500 μL of NP‐40 Lysis Buffer, incubated on ice for 10 min, then centrifuged at 21,000 x g for 10 min at 4°C. The supernatants were gently transferred into a new 1.5‐mL tube and saved as the “mitochondrial extract” (Mito).

WCE, Cyto and Mito fractions were treated with 5 mg/mL of RNase A at 37°C for 1.5 h and subsequently with 20 mg/mL of Proteinase K at 55°C for 1 h, followed by adding 400 μL of phenol/chloroform/isoamyl alcohol, vortexed vigorously for 1 min and spun at 21,000*g* for 5 min at RT. The upper aqueous phase was transferred into a new tube, added an equal volume of chloroform/isoamyl alcohol, vortexed and spun. Again, the upper aqueous phase was transferred into a new tube, followed by adding 7.5 M NH_4_OAc for a final concentration of 0.75 M, 20 μg glycogen and mixed completely. 2.5 vol of 100% ethanol was added and mixed well, followed by incubating at −80°C for 1 h (or −20°C overnight) to precipitate mtDNA. mtDNA is finally pelleted by spinning at max speed in a 4°C for 20 min in an Eppendorf centrifuge. mtDNA were washed 3 times with 300 μL of 95% EtOH And resuspend in 20–100 μL of nuclease‐free water. mtDNAs were subjected to SYBR Green‐based qPCR for the quantification. Primers qPCR for mtDNAs and nuclear genomic DNAs are shown in Table S2.

### 4.10 Detection of Soluble and Insoluble Proteins in Thoraces and Heads of Flies

As previously described (He et al. 2019), 60 male flies per group were dissected. Thoraces and heads were homogenized in 1% Triton X‐100 in PBS containing a protease inhibitor cocktail and phosphatase inhibitors, followed by centrifugation at 12,000 g for 15 min at 4°C. The supernatants were collected as the Triton X‐100 soluble fraction. Cell debris was rinsed with 1% Triton X‐100 in PBS three times and then homogenized in 2× SDS lysis buffer (63 mM Tris HCl, pH 6.8, 2% SDS, 10% glycerol, and protease inhibitor cocktail). After centrifugation at 15,000 g for 15 min at room temperature, supernatants (Triton X‐100 insoluble fraction) were collected. Both fractions were separated on polyacrylamide gels and immunoblotted with anti‐ubiquitin (1:1000) and anti‐β‐actin (1:5000) antibodies.

### 4.11 Lifespan Analysis

Flies were anesthetized with light CO_2_ exposure and placed in vials at a density of 30 males per vial. They were maintained in a temperature‐controlled incubator with a 12‐h light/dark cycle at 25°C. Every 2–3 days, flies were moved to fresh food vials. Their survival status was monitored and documented.

### 4.12 *Drosophila* Climbing Assay

The climbing assay was conducted essentially as described (He et al. 2019; Zhang et al. 2016). In brief, groups of ten 5‐day‐old and 30‐day‐old male flies were anesthetized with CO_2_ and transferred into transparent plastic vials measuring 25 cm in length and 1.5 cm in diameter. Flies were then rested for 30 min. Flies were tapped down to the bottom of the vial. Time required for five flies to climb up to a 10‐cm finish line was recorded. The climbing index is reported as speed (cm/s). The assay was repeated three times for each group with 5‐min intervals. At least 100 flies of each genotype were tested. The mean climbing time for each was calculated.

### 4.13 ATP Assay

ATP levels were measured using a commercial kit (FF2000, Promega, Madison, WI) (Han et al. 2020; Zhang et al. 2016). Briefly, lysates from five thoraces of flies were prepared for each experiment. Samples were mixed with luminescent solution. The luminescence was measured by a luminometer (Berthold Technologies, Bad Wildbad, Germany). Results were normalized to protein contents.

### 4.14 Transmission Electron Microscopy (TEM)

As previously described (Han et al. 2020), muscles dissected from flies were fixed in a solution containing paraformaldehyde and glutaraldehyde, followed by post‐fixation in osmium tetroxide (19,150, Electron Microscopy Sciences, Hatfield, PA). Subsequently, samples were dehydrated in a graded ethanol series and embedded in Epon resin (45,345, Sigma‐Aldrich, St. Louis, USA). After polymerization, ultrathin sections of 70 nm thickness were prepared using a diamond knife on an ultramicrotome (Leica, Wetzlar, Germany). Sections were stained with uranyl acetate and lead citrate for enhanced contrast. Digital images were acquired using a Tecnai G2 Spirit transmission electron microscope (FEI) equipped with an Eagle 4 k HS digital camera (Hitachi High‐Tech, Marunouchi, Japan).

### 4.15 Detection of Neurodegeneration in *Drosophila* Whole‐Brain Mounts

Detection of neurodegeneration in *Drosophila* whole‐brain mounts was performed as previously described (Behnke et al. 2021). Briefly, fly brains were dissected and fixed in 4% paraformaldehyde containing 0.5% Triton X‐100 at 20°C–23°C with nutation for 20 min. Brains were incubated 16–24 h at 4°C with phalloidin (1:100) and DAPI (1:1000) diluted in 0.5% PBST. Fly brains were washed 4 times with 0.5% PBS‐T with nutation at 20°C–23°C for 15 min each. Finally, fly brains were washed in PBS at 20°C–23°C for 30 min with nutation to remove residual detergents. Brains were mounted with the anterior side facing up using Fluoromount.

### 4.16 RNA Extraction, Sequencing and Analysis

As previously described (Wang et al. 2024), total RNA was extracted from tissues using a Trizol‐based method (15,596,026, Thermo Fisher Scientific). The concentration and integrity of RNA concentration and integrity were assayed using a NanoDrop spectrophotometer (Thermo Fisher Scientific) and an Agilent 2100 Bioanalyzer (Agilent, Santa Clara, CA), respectively. Poly(A) RNAs were isolated from total RNA using oligo‐dT magnetic beads (S1419S, New England Biolabs, Ipswich, MA). Poly(A) RNAs were fragmented into approximately 200 bp fragments. First‐strand cDNA synthesis was performed using random hexamers, followed by second‐strand synthesis to generate double‐stranded cDNA. The cDNA library was sequenced on an Illumina HiSeq platform (San Diego, CA), yielding 150 bp paired‐end reads. Raw sequencing data were subjected to quality control analysis using FastQC and aligned to the reference genome (UCSC *Drosophila* genome version dm6) using alexdobin/STAR (Version 2.4, Spliced Transcripts Alignment to a Reference Alexander Dobin). Gene expression levels were quantified via featureCounts‐2.0.3 (featureCounts is available under GNU General Public License as part of the Subread (https://subread.sourceforge.net) or Rsubread (https://www.bioconductor.org) software packages). Differential gene expression analysis was performed using DESeq2 (Version 3.19, Bioconductor).

### 4.17 Real‐Time PCR

As previously described (Wang et al. 2024), total RNA isolated using Trizol reagent (15,596,026, Thermo Fisher Scientific, Waltham, MA) was converted to cDNA with the Verso cDNA Kit (K1691, Thermo Fisher Scientific, Waltham, MA) according to the manufacturer's instructions. 2 × SYBR Green qPCR Master Mix (K0251, Thermo Fisher Scientific, Waltham, MA) was used for quantitative real‐time PCR amplification with a CFX96 Real‐Time PCR Detection System (Bio‐Rad, Hercules, CA) and corresponding software (Applied Biosystems, Thermo Fisher Scientific, Waltham, MA). PCR was performed with 1 cycle at 50°C for 2 min and 95°C for 10 min, followed by 40 cycles of 95°C for 15 s and 60°C for 1 min. Gene expression was normalized to Rp49. Relative mRNA levels were calculated based on the comparative Ct method. Primers for AttA, CecA1, and CecC amplification are shown in Table S2.

### 4.18 N‐Acetyl‐L‐Cysteine (NAC) and Antibiotic Treatments

NAC (ST1546, Beyotime, Shanghai, China) was dissolved in solvents recommended by suppliers and subsequently mixed with *Drosophila* food. The final concentration of NAC was adjusted to 15 mM. Control groups were prepared by mixing the respective solvents. Embryos of fruit flies were produced on the surface of food containing 15 mM NAC and maintained on food containing NAC.

Antibiotic treatments followed the procedure described previously (Zhou et al. 2021). An antibiotic mixture (150 μg/mL Ampicillin, 150 μg/mL Metronidazole, and 75 μg/mL Tetracycline) was used in this study. To raise flies under axenic conditions, embryos were first collected on apple juice plates. Embryos (< 12 h) were then washed with PBS, rinsed in 70% ethanol, and bleached with 3% sodium hypochlorite for 2 min, followed by washing three times with sterile water. Axenic embryos were transferred to the autoclaved medium and maintained at 25°C. Offspring of flies were cultured on sterile food for longevity analysis. The germ‐free (GF) conditions were confirmed by plating fly gut homogenates onto agar plates.

### 4.19 Whole‐Mount Brain Immunohistochemistry for SA‐β‐Gal Activity

SA‐β‐Gal (9860, Cell Signaling Technology, Boston, MA) activity of whole‐brain mounts was detected as previously described (Byrns et al. 2024; Debacq‐Chainiaux et al. 2009). Briefly, adult fly brains were dissected in cold PBS and fixed in 2% paraformaldehyde (vol/vol) and 0.2% glutaraldehyde (vol/vol) at room temperature for 30 min. Brains were washed in PBS, incubated in 200 μL of β‐Galactosidase Staining Solution (9860, CST, Boston, MA) at 37°C in dark with shaking (300 rpm) for 12–24 h. Brains were washed in PBS followed by mounting with the anterior side facing upward using Fluoromount. Images were collected on a Leica M205 FCA microscope (Wetzlar, Germany) and quantified in ImageJ (Version 1.54).

### 4.20 Statistical Analysis

Statistical analysis was performed using Prism 9 software (GraphPad, La Jolla, CA). Two‐tailed Student's *t*‐test was used to determine the significance of difference between two groups. Statistical significance between multiple groups was analyzed with one‐way ANOVA followed by Tukey's post hoc test. Comparisons between treatment groups and their controls was assessed using a multiple‐way ANOVA with Dunnett's tests. All error bars represent SEM. Quantitation was performed in a double‐blinded manner.

## Author Contributions

Z.Z. conceived, initiated, designed and supervised the project. Z.D., H.H. and Z.Z. performed experiments and data analysis. C.W., W.Z., Z.W., D.Z., Y.C., Y.P., Z.Z., K.Y., and R.W. provided technical support. Z.Z., Z.D. and H.H. wrote the manuscript. All authors have read and approved the article.

## Funding

This work was supported by grants from the National Natural Science Foundation of China (22494703, 22494700, 82201412, and 31330031), the Project of Changping Laboratory (2025B‐07‐40), the Science and Technology Innovation Program of Hunan Province (2021SK1010), the National Key R&D Program of China (2024YFA1804000), and the Department of Science and Technology of Hunan Province (2022CB1004).

## Conflicts of Interest

The authors declare no conflicts of interest.

## Supporting information


**Table S1:** Lifespan of flies expressing *h*BNIP3 in different tissues.
**Table S2:** Primers used in this study.
**Figure S1:** Validation of mito‐SRAI for detecting mitophagy in *Drosophila*.
**Figure S2:** Performance comparison of mitophagy reporters mito‐SRAI, mito‐QC and mito‐Keima in vivo.
**Figure S3:** Verification of exogenous mitophagy protein expression in *Drosophila* muscle and mitochondrial morphological abnormalities in aged BNIP3 knockdown flies.
**Figure S4:** Effects of *BNIP3* expression in different fly tissues on lifespan of *Drosophila*.
**Figure S5:** BNIP3 suppresses the activation of age‐associated activation of Relish signaling in IFMs.
**Figure S6:** BNIP3 regulated Relish signaling is *Atg1*‐dependent.
**Figure S7:** *BNIP3*‐induced lifespan extension is independent of the microbial environment.
**Figure S8:** Knockdown of Relish‐related *DptA*, *AttA*, *AttC*, *PGRP‐SC1b*, *PGRP‐LE*, *Tak1*, and *Tak2* genes had little effect on *Drosophila* lifespan.
**Figure S9:** BNIP3 inhibits mtDNA release through a STING/Eya‐independent mechanism in aging flies.
**Figure S10:** ROS clearance rescues the shortened lifespan of *BNIP3* ^*Δ*^ flies.
**Figure S11:** Knockdown of *CecA1* or *CecC* in skeletal muscle suppresses age‐dependent neurodegeneration in *Drosophila*.

## Data Availability

All data are available in the main text or Supporting Information. Datasets generated during and/or analyzed during the current study are available from the corresponding author upon reasonable request.
