# Supplementary material for: BNIP3‐Dependent Mitophagy Non‐Autonomously Regulates Systemic Aging via NF‐κB Suppression in Drosophila
Source: Aging Cell. 2026 May 13;25(5):e70539. doi: 10.1111/acel.70539 (PMC13171292; doi:10.1111/acel.70539)
Supplement: Supplementary file 1 — Table S1: Lifespan of flies expressing hBNIP3 in different tissues. Table S2: Primers used in this study. Figure S1: Validation of mito‐SRAI for detecting mitophagy in Drosophila. Figure S2: Performance comparison of mitophagy reporters mito‐SRAI, mito‐QC and mito‐Keima in vivo. Figure S3: Verification of exogenous mitophagy protein expression in Drosophila muscle and mitochondrial morphological abnormalities in aged BNIP3 knockdown flies. Figure S4: Effects of BNIP3 expression in different fly tissues on lifespan of Drosophila. Figure S5: BNIP3 suppresses the activation of age‐associated activation of Relish signaling in IFMs. Figure S6: BNIP3 regulated Relish signaling is Atg1‐dependent. Figure S7: BNIP3‐induced lifespan extension is independent of the microbial environment. Figure S8: Knockdown of Relish‐related DptA, AttA, AttC, PGRP‐SC1b, PGRP‐LE, Tak1, and Tak2 genes had little effect on Drosophila lifespan. Figure S9: BNIP3 inhibits mtDNA release through a STING/Eya‐independent mechanism in aging flies. Figure S10: ROS clearance rescues the shortened lifespan of BNIP3 Δ flies. Figure S11: Knockdown of CecA1 or CecC in skeletal muscle suppresses age‐dependent neurodegeneration in Drosophila. [file ACEL-25-e70539-s001.docx]

**Supplementary Table:**

**Supplementary Table S1.** Lifespan of flies expressing *h*BNIP3 in different tissues.

|  | Median survival (days) | | p-value | n | |
| --- | --- | --- | --- | --- | --- |
| Cross with | *w^1118^* | *h*BNIP3 OE | *h*BNIP3 OE vs *w^1118^* | *w^1118^* | *h*BNIP3 OE |
| Mhc-gal4 | 53 | 64 | <0.0001 | 553 | 464 |
| Cg-gal4 | 47 | 53 | <0.0001 | 286 | 283 |
| Elav-gal4 | 52 | 38 | <0.0001 | 282 | 292 |
| Repo-gal4 | 61 | 66 | 0.0021 | 267 | 288 |
| Myo1a-gal4 | 46 | 46 | 0.4370 | 220 | 286 |
| Da-gal4 | Lethal | | | | |

*w^1118^*: wildtype control flies. *h*BNIP3 OE: flies overexpressing *h*BNIP3.

**Supplementary Table S2.** Primers used in this study

| **Name** | **Primer sequences** |
| --- | --- |
| Common sequence-Fw  Common sequence-Rev | 5ʹ-CGCAAATGGGCGGTAGGCGTG-3ʹ  5ʹ- TAG AAGGCACAGTCGAGG-3ʹ |
| AttA qPCR-Fw  AttA qPCR-Rev | 5ʹ-ACAATGTGGTGGGTCAGGTT-3ʹ  5ʹ-CCAGATTGTGTCTGCCATTG-3ʹ |
| CecA1 qPCR-Fw  CecA1 qPCR-Rev | 5ʹ-TCTTCGTTTTCGTCGCTCTC-3ʹ  5ʹ-CTTGTTGAGCGATTCCCAGT-3ʹ |
| CecC qPCR-Fw  CecC qPCR-Rev | 5ʹ-TGTAAGCTAGTTTATTTCTATGG-3ʹ  5ʹ-GATGAGCCTTTAATGTCC-3ʹ |
| Rp49 qPCR-Fw  Rp49 qPCR-Rev | 5ʹ-GCCGCTTCAAGGGACAGTATCT-3ʹ  5ʹ-AAACGCGGTTCTGCATGAG-3ʹ |
| Atg1 qPCR-Fw  Atg1 qPCR-Rev | 5ʹ-GCGACTTGGCGGATTATCTG-3ʹ  5ʹ-TTTCGATGGAGCTGGCAATG-3ʹ |
| BNIP3 qPCR-Fw  BNIP3 qPCR-Rev | 5ʹ-TGATAGCCCCAAGAGTCCAC-3ʹ  5ʹ-TCTTCATTGCCTGCATTCTG-3ʹ |
| mtDNA qPCR-Fw  mtDNA qPCR-Rev | 5ʹ-CTTTTATCCCCCTATTAAGAG-3ʹ  5ʹ-GAAGCTTCTGTAGATATTAAATTATTA-3ʹ |
| nuclear genomic qPCR-Fw  nuclear genomic qPCR -Rev | 5ʹ-GCCAATGATAAGCGCCTTGAATATAA-3ʹ  5ʹ-CTGGTGCCAGTAACCGTTGT-3ʹ |

**Supplementary Figures and Figure Legends:**

**
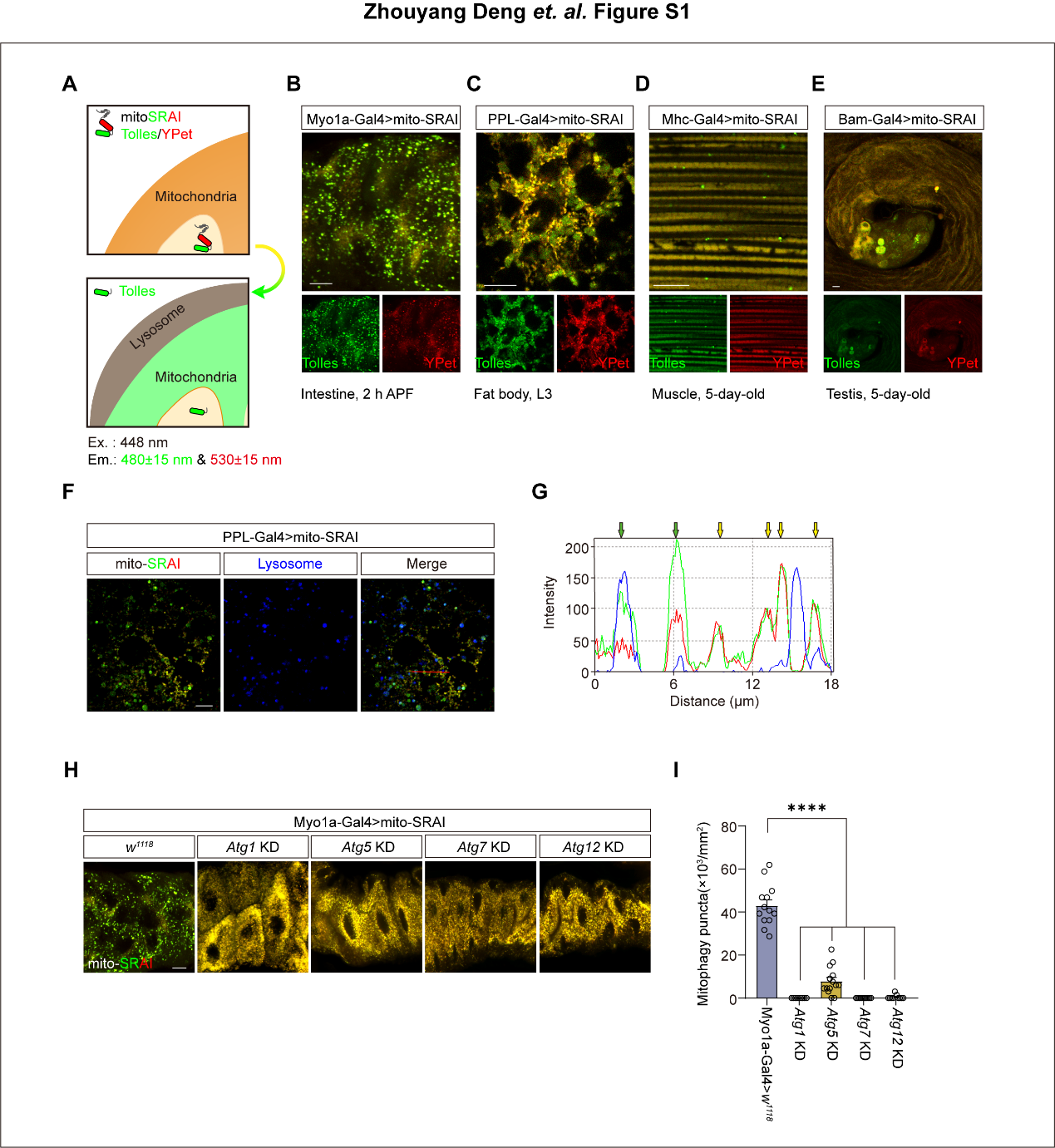
**

**Supplementary Figure S1. Validation of mito-SRAI for detecting mitophagy in *Drosophila***

1. Schematic illustration of mito-SRAI imaging. Mito-SRAI was originally developed by Katayama et al(Katayama et al., 2020). Mito-SRAI is a tandem fusion of YPet and TOLLES that is targeted to the mitochondrial matrix. When mitochondria are delivered to acidic lysosomes, YPet (red) is degraded and TOLLES (green) is retained. Mito-SRAI is a single-excitation dual-emission imaging sensor, which uses an excitation filter (448 nm) and two emission filters (480 ± 15 nm for TOLLES and 530 ± 15 nm for YPet). The TOLLES/YPet fluorescent ratio puncta represent degrading mitochondria.

**(B-E)** Confocal microscopic analysis of mitophagy by mito-SRAI in different fly tissues. Myo1a-Gal4>UAS-mito-SRAI to determine mitophagy in the Intestine 2 hr after puparium formation (2 h APF), Bar = 10 μm **(B)**; PPL-Gal4>UAS-mito-SRAI to determine mitophagy in third larval instar (L3) fat body tissues after 4h starvation, Bar = 10 μm **(C)**; Mhc-Gal4>UAS-mito-SRAI to determine mitophagy in adult indirect flight muscles, Bar = 10 μm **(D)**; and Bam-Gal4>UAS-mito-SRAI to determine mitophagy in adult fly testes, Bar = 10 μm **(E)**.

**(F and G)** Confocal live imaging of third larval instar (L3) fat body tissues. Wildtype flies were processed for a 4 h starvation. Note that mito-SRAI puncta were colocalized with lysosomal LysoTracker Deep Red (blue). Red line indicates region for colocalization-quantification **(F)**. Quantification of fluorescence intensity for both mito-SRAI and LysoTracker in *Drosophila* fat body was shown **(G)**.

**(H and I)** Mito-SRAI in intestines 2 h APF of flies with autophagy gene knockdown. Wildtype flies (Myo1a-Gal4*>w^1118^*) and flies expressing *Atg1* RNAi (*Atg1* KD), *Atg5* RNAi (*Atg5* KD), *Atg7* RNAi (*Atg7* KD) or *Atg12* RNAi (*Atg12* KD) were analyzed. Bar = 10 μm **(H)**. Quantification of mito-SRAI labelled mitophagy. n =13 (Myo1a-Gal4>*w^1118^*), 9 (*atg1* KD), 14 (*atg5* KD), 11 (*atg7* KD), 10 (*atg12* KD) guts **(I)**. One-way ANOVA followed by Tukey’s test. ****p < 0.0001. Data are from at least three biological replicates and presented as mean ± SEM.


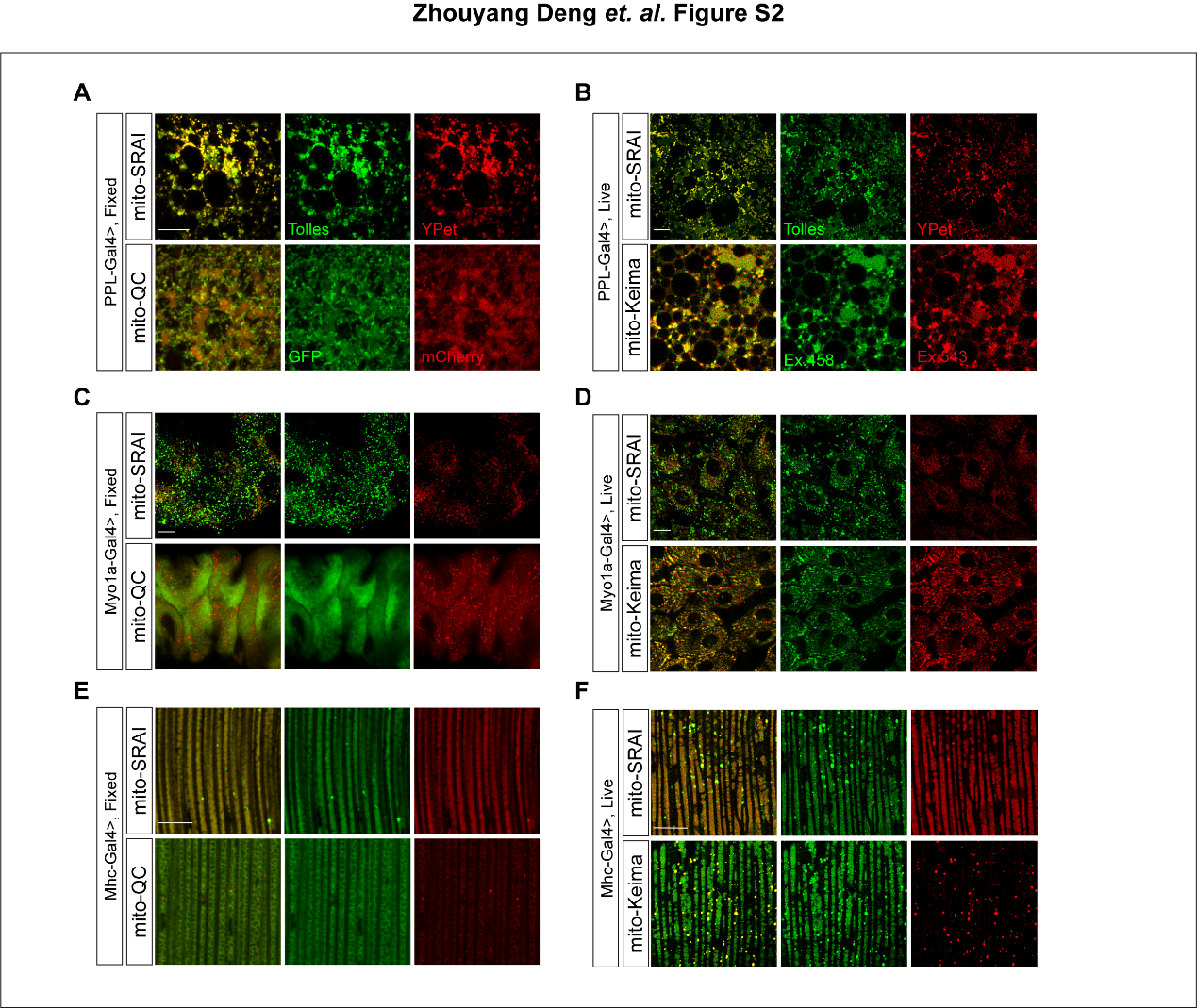


**Supplementary Figure S2. Performance comparison of mitophagy reporters mito-SRAI, mito-QC and mito-Keima *in vivo***

**(A, C, E)** Confocal images of mito-SRAI and mito-QC in various fixed fly tissues. Third larval instar (L3) fat body tissue after 4 h starvation **(A)**, intestine 2 h APF **(C)** or adult fly muscles **(E)** derived from flies expressing either mito-SRAI or mito-QC were imaged. Bar = 10 μm. Note that mitophagy (green puncta) is clearly detected in fixed tissues using mito-SRAI.

**(B, D, F)** Confocal images of mito-SRAI and mito-Keima in various living fly tissues. third-instar larval (L3) fat body tissues after 4 h starvation **(B)**, intestine 2 h APF **(D)** or adult indirect flight muscles **(F)** derived from flies expressing either mito-SRAI or mito-Keima were imaged. Bar = 10 μm.


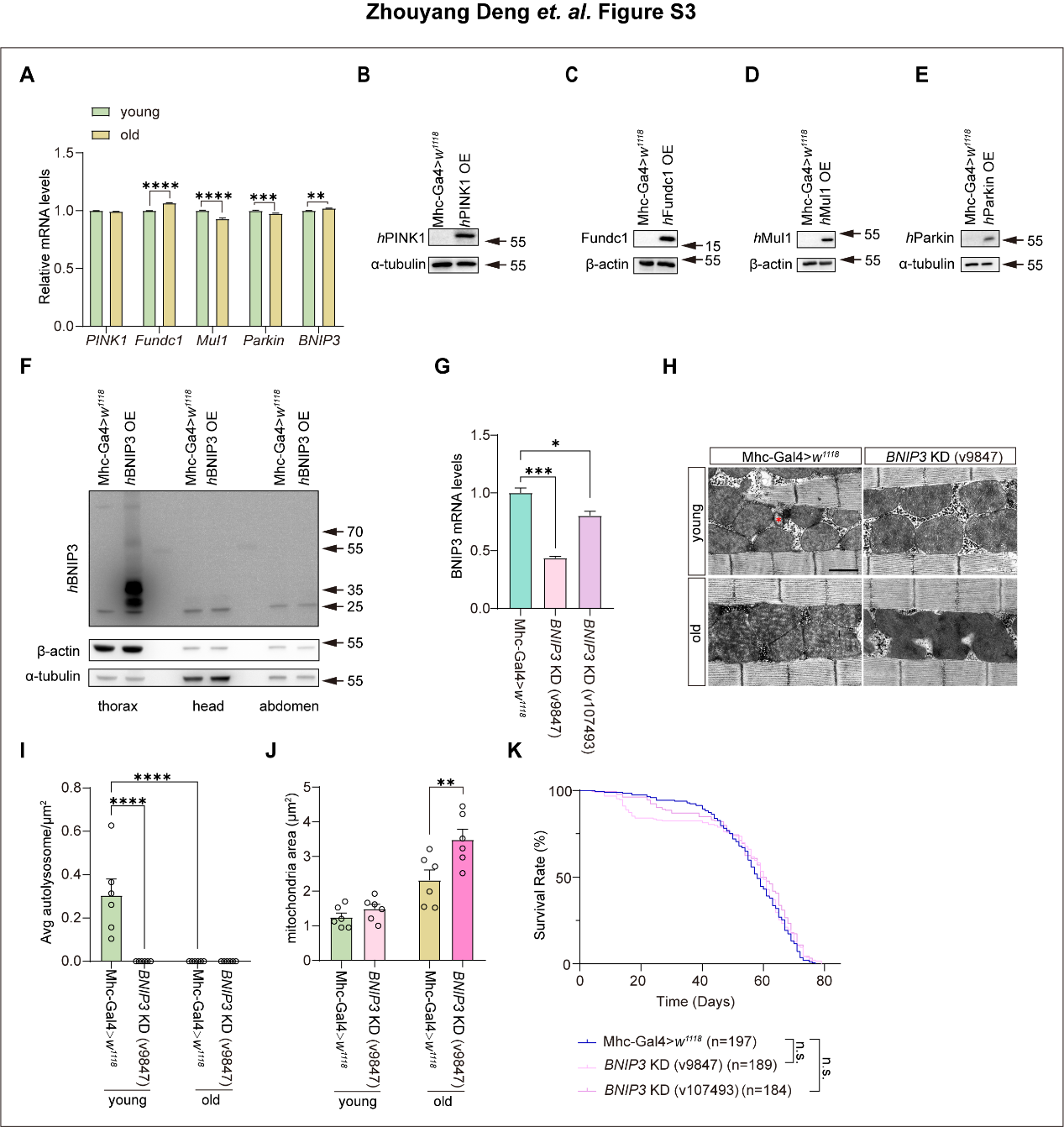


**Supplementary Figure S3. Verification of exogenous mitophagy protein expression in *Drosophila* muscle and mitochondrial morphological abnormalities in aged BNIP3 knockdown flies**

**(A)** Expression of PINK1, Fundc1, Mul1, Parkin, and BNIP3 in *Drosophila* thoraces, based on RNA-seq data from young (5-day-old) and old (30-day-old) wild-type flies. Statistical significance was determined by two-way ANOVA followed by Tukey’s test. ****p < 0.0001, ***p < 0.001, **p < 0.01. Data are presented as mean ± SEM.

**(B-E)** Immunoblotting analysis of exogenous mitophagy proteins in young (5-day-old) flies. Thoraces from wildtype flies (Mhc-Gal4*>w^1118^*) and flies overexpressing *h*PINK1 (**B**, *h*PINK1 OE), *h*Fundc1 (**C**, *h*Fundc1 OE), *h*Mul1 (**D**, *h*Mul1 OE), *h*Parkin (**E**, *h*Parkin OE) were analyzed. β-actin or α-tubulin is detected as loading control.

**(F)** Immunoblotting analysis of exogenous human BNIP3 levels in different tissues of young (5-day-old) flies. Thoraces, heads and abdomens from wildtype flies (Mhc-Gal4*>w^1118^*) and flies overexpressing *h*BNIP3 (*h*BNIP3 OE) were analyzed. Both β-actin and α-tubulin were detected as loading controls.

**(G)** Verification of *BNIP3* RNAi efficiency in *Drosophila*. Thoraces derived from flies with *BNIP3* RNAi (*BNIP3 KD*, v9847, v107493) and wildtype control flies (Mhc-Gal4>*w^1118^*) were analyzed by qPCR. Unpaired Student’s t-test. ***p < 0.001, *p < 0.05. Data are presented as mean ± SEM from at least three biological replicates.

**(H-J)** Representative TEM images of mitochondria in IFMs. Mitochondrial morphology in IFMs was analyzed under TEM. IFMs derived from young (5-day-old) and old (30-day-old) flies, including wildtype flies (Mhc-Gal4*>w^1118^*) and flies with muscle-specific *BNIP3* RNAi (*BNIP3* KD, v9847) were analyzed. Red asterisk indicates autolysosome. Bar = 1 μm **(H)**. Quantification of autolysosomes **(I)**. n = 6 IFMs for each group. Quantification of mitochondria area **(J)**. n = 6 IFMs for each group. Two-way ANOVA followed by Tukey’s test. ***p < 0.001. Data are presented as mean ± SEM from at least three biological replicates.

**(K)** Lifespan analysis. Wildtype flies (Mhc-Gal4*>w^1118^*) and flies with muscle-specific *BNIP3* RNAi (*BNIP3* KD, v9847, v107493) were assayed for lifespan. The number of flies (n) is indicated in the figure. Statistical analysis was done using Log-rank test. n.s., no significance.

**
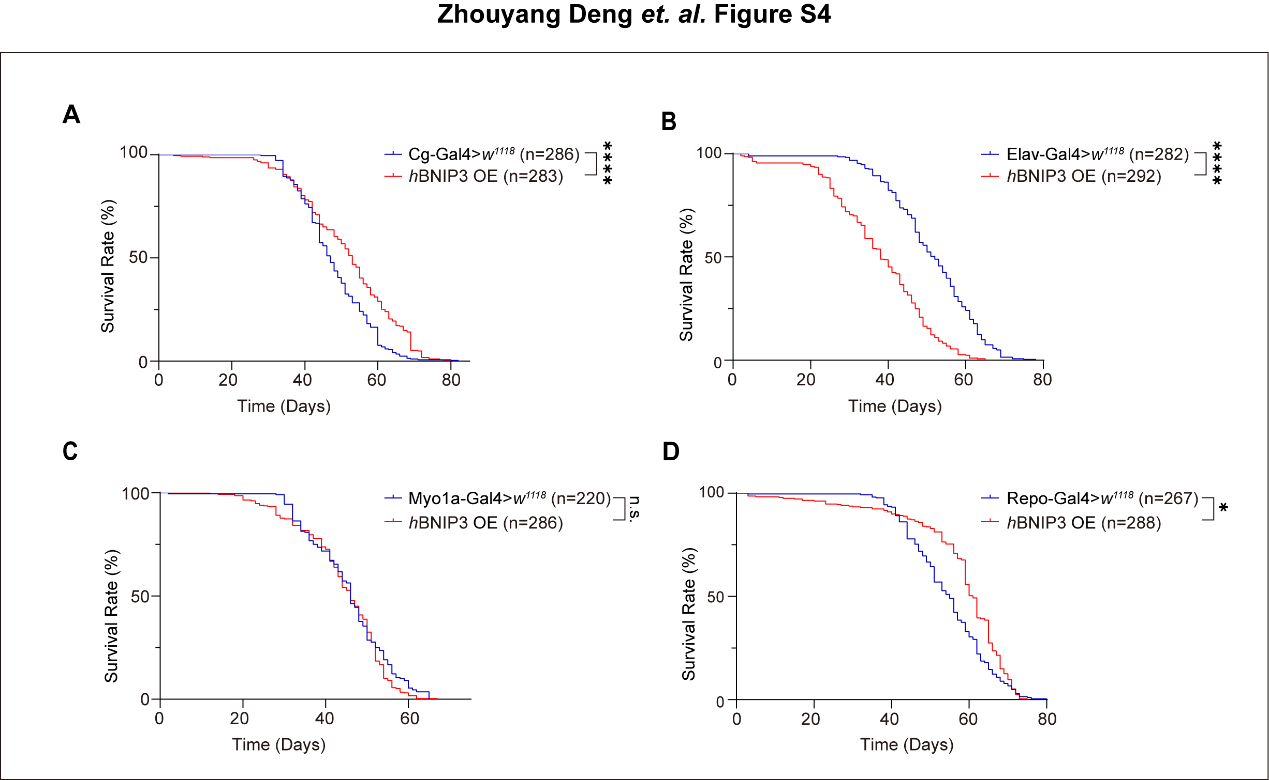
**

**Supplementary Figure S4. Effects of *BNIP3* expression in different fly tissues on lifespan of *Drosophila***

**(A-D)** The lifespan of flies with either fat body specific expression **(A)**, neuronal expression **(B)**, enterocytes (ECs) expression (midgut intestinal epithelium tissue) **(C)**, or glial cell expression **(D)** of *h*BNIP3 were analyzed. The number of flies (n) is indicated in the figure. Log-rank test. ****p < 0.0001, *p < 0.05, n.s., no significance. Note that neuronal expression of *h*BNIP3 markedly shortened fly lifespan.

**
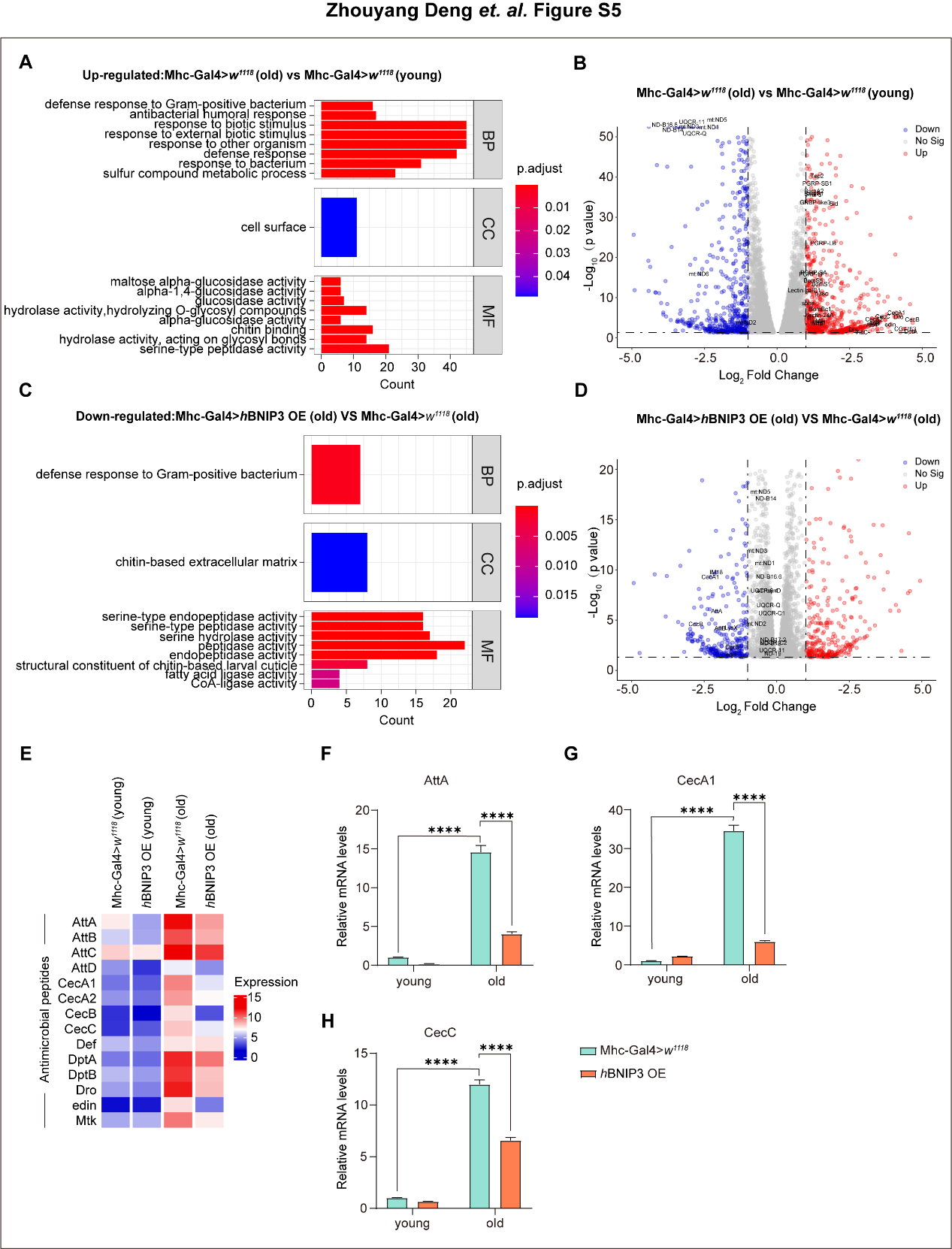
**

**Supplementary Figure S5.** **BNIP3 suppresses the activation of age-associated activation of Relish signaling in IFMs**

**(A and C)** GO enrichment analysis of the up-regulated genes in thoraces of old (30-day-old) versus young (5-day-old) wildtype flies (Mhc-Gal4*>w^1118^*) **(A)** and down-regulated genes in thoraces of old flies with muscle *h*BNIP3 overexpression (*h*BNIP3 OE) compared to old wildtype flies (Mhc-Gal4*>w^1118^*) **(C)**. The most significant categories of up-regulated genes are shown with adjusted P-values of <0.05 and logFC >1. The most significant categories of down-regulated genes are shown with adjusted P-values of <0.05 and logFC <-1. BP: Biological Process, CC: Cell Component, MF: Molecular Function.

**(B and D)** Volcano plot of the whole-transcriptome RNA-seq data of thoraces derived from old (30-day-old) compared to young (5-day-old) wildtype flies (Mhc-Gal4*>w^1118^*) **(B)** and thoraces derived from old flies overexpressing *h*BNIP3 (*h*BNIP3 OE) compared to old wildtype flies (Mhc-Gal4*>w^1118^*) **(D)**. Genes have a threshold of two-fold changes with adjusted P-values of <0.05 and logFC >1 or <-1 are considered as significant. No significant genes are presented as gray dots, whereas up-regulated and down-regulated genes with p < 0.05 are highlighted with red and blue dots, respectively. A two-tailed moderated t-test was performed. Data are from three independent experiments.

**(E)** Expression of the selected AMP genes in the RNA-seq data derived from *Drosophila* thoraces wildtype flies (Mhc-Gal4*>w^1118^*) and overexpressing *h*BNIP3 (*h*BNIP3 OE)*.* Young, 5-day-old; old, 30-day-old.

**(F-H)** qPCR analysis of AttA, CecA1 and CecC expression. Thoraces derived from young (5-day-old) and old (30-day-old) flies, including wildtype flies (Mhc-Gal4*>w^1118^*) and overexpressing *h*BNIP3 (*h*BNIP3 OE). Two-way ANOVA followed by Tukey’s test. ****p < 0.0001. Data are from at least three biological replicates and presented as mean ± SEM.

**
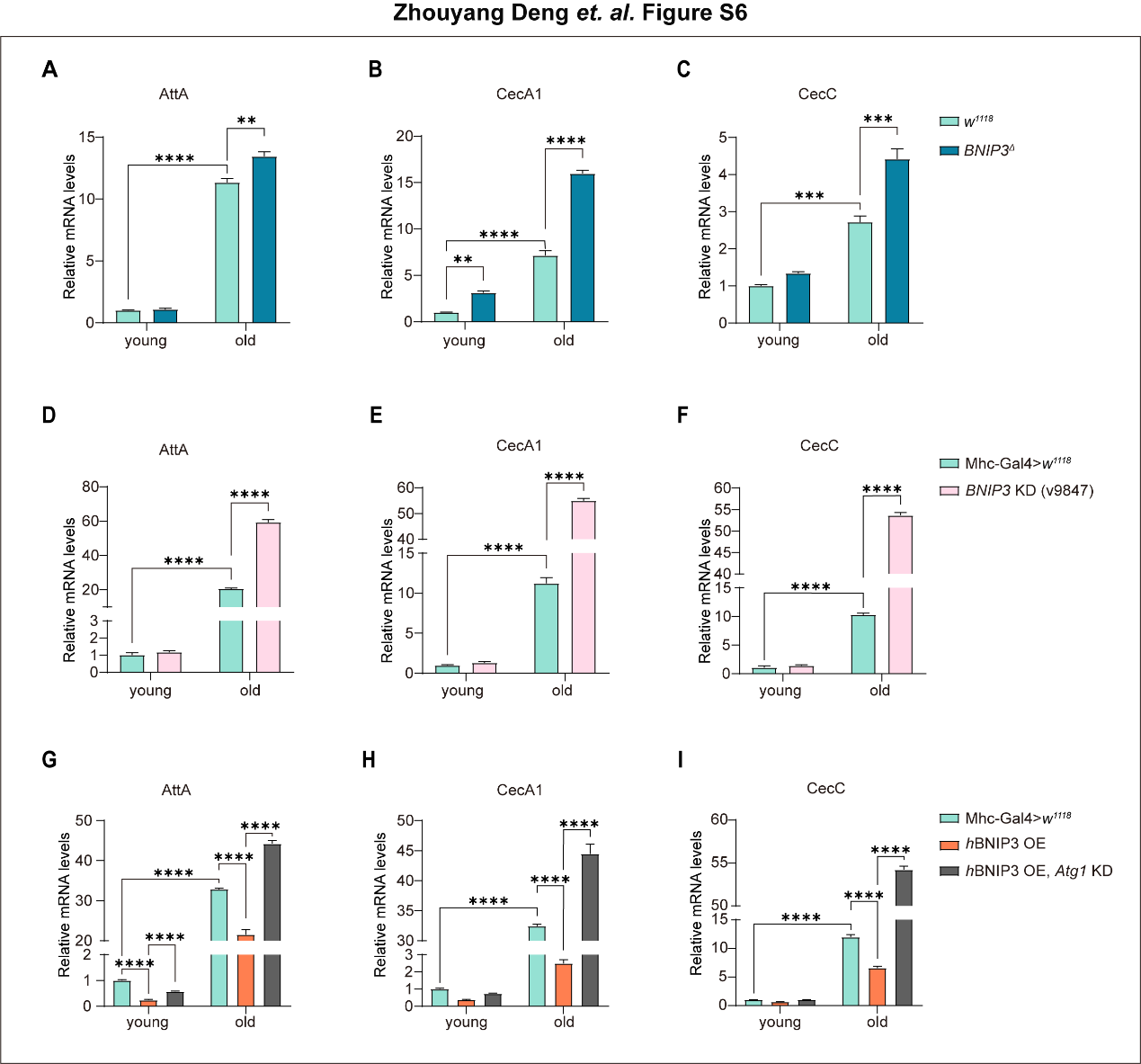
**

**Supplementary Figure S6. BNIP3 regulated Relish signaling is *Atg1*-dependent**

**(A-C)** Expression of AttA, CecA1 and CecC was monitored by qPCR. Thoraces derived from wildtype *w^1118^* flies *and BNIP3^Δ^* mutant flies were analyzed. Young:5-day-old; old: 30-day-old. Two-way ANOVA followed by Tukey’s test. ****p < 0.0001, ***p < 0.001, **p < 0.01. Data are from at least three biological replicates and presented as mean ± SEM. Note that expression of AttA, CecA1 and CecC is significantly increased in *BNIP3^Δ^* mutant flies.

**(D-F)** Aging-induced expression of AttA, CecA1 and CecC was further enhanced with *BNIP3* knockdown. Thoraces derived from flies with muscle-specific *BNIP3* RNAi (BNIP3 KD, v9847) and wildtype control flies (Mhc-Gal4>*w^1118^*) were analyzed by qPCR. Young, 5-day-old; old, 30-day-old. Two-way ANOVA followed by Tukey’s test. ****p < 0.0001. Data are from at least three biological replicates and presented as mean ± SEM.

**(G-I)** Suppression of AttA, CecA1 and CecC expression by *BNIP3* was inhibited by Atg1 knockdown. Thoraces of wildtype control flies (Mhc-Gal4>*w^1118^*) and flies overexpressing *h*BNIP3 (*h*BNIP3 OE) and expressing combination of *h*BNIP3 and *Atg1* RNAi (*h*BNIP3 OE, *Atg1* KD) were analyzed. Young, 5-day-old; old, 30-day-old. Two-way ANOVA followed by Tukey’s test. ****p < 0.0001. Data are from at least three biological replicates and presented as mean ± SEM.


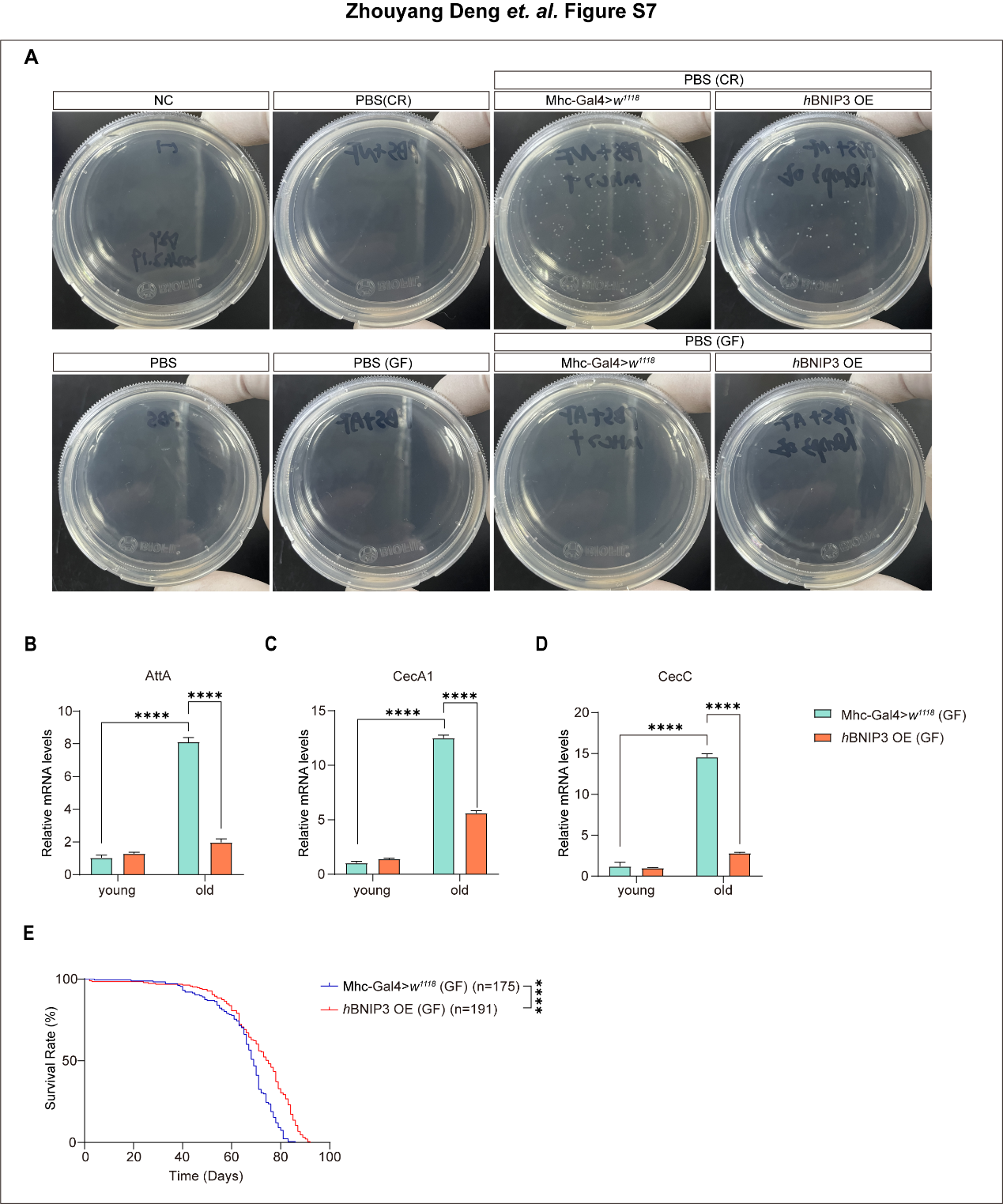


**Supplementary Figure S7. *BNIP3*-induced lifespan extension is independent of the microbial environment**

1. Validation of gut commensal microbes in conventionally reared (CR) and germ-free (GF) flies. Guts derived from wildtype control flies (Mhc-Gal4>*w^1118^*) flies and flies overexpressing *h*BNIP3 (*h*BNIP3 OE) treated with or without antibiotic food were dissected and homogenates were plated on Agar plates. Blank Agar plate, sterile PBS and fruit fly food with or without antibiotics were used as negative controls.

**(B-D)** qPCR analysis of AttA, CecA1 and CecC expression. Thoraces derived from young (5-day-old) and old (30-day-old) flies, including GF wildtype flies (Mhc-Gal4*>w^1118^*, GF) and GF flies overexpressing *h*BNIP3 (*h*BNIP3 OE, GF). Two-way ANOVA followed by Tukey’s test. ****p < 0.0001. Data are from at least three biological replicates and presented as mean ± SEM.

**(E)** Lifespan analysis of GF flies expressing *h*BNIP3. GF wildtype flies (Mhc-Gal4*>w^1118^*, GF) and GF flies overexpressing *h*BNIP3 (*h*BNIP3 OE, GF) were assayed for lifespan. n number is indicated in the figure. Log-rank test. ****p < 0.0001.


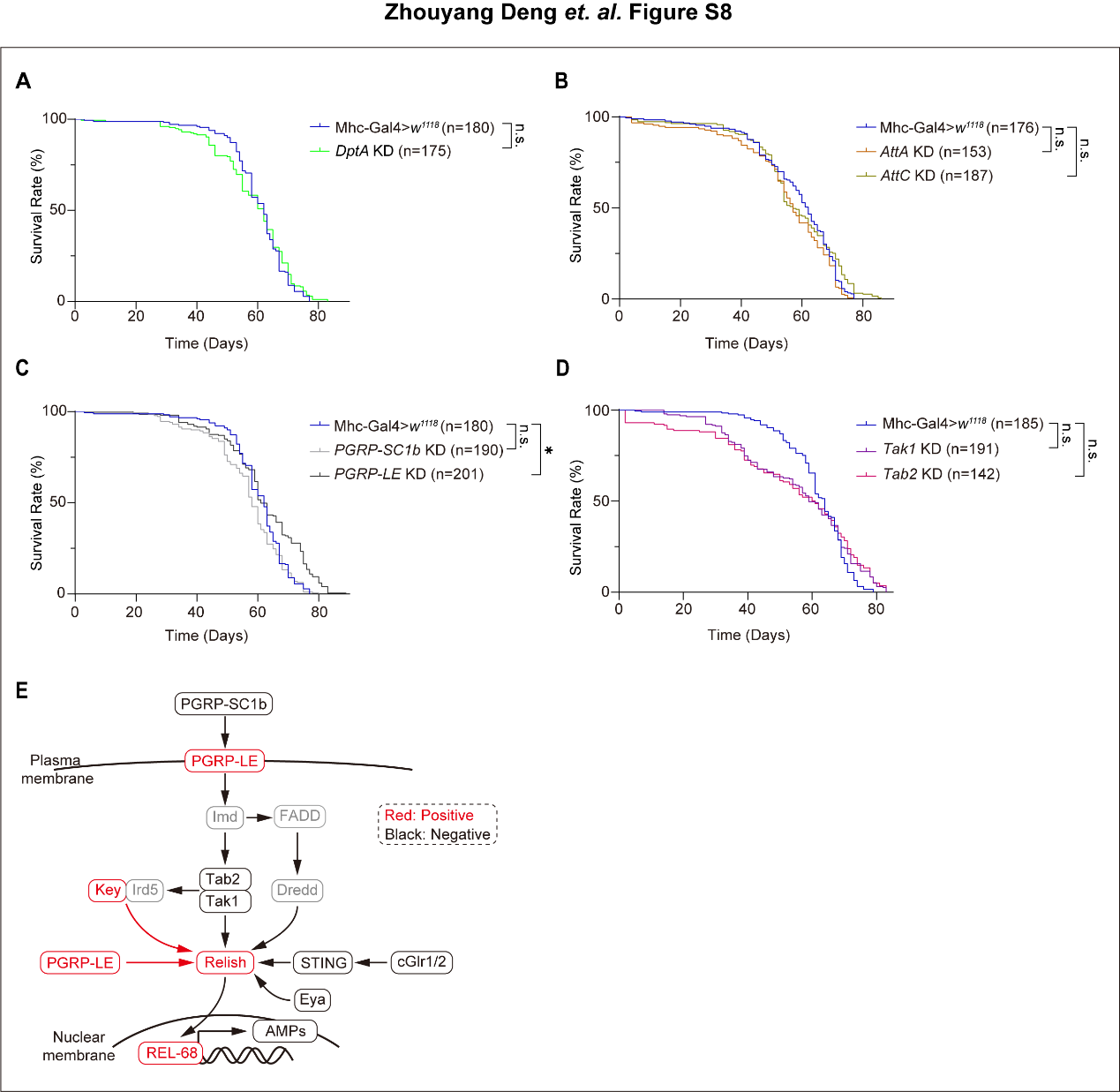


**Supplementary Figure S8. Knockdown of Relish-related *DptA*, *AttA*, *AttC*, *PGRP-SC1b*, *PGRP-LE*, *Tak1*, and *Tak2* genes had little effect on *Drosophila* lifespan**

**(A-D)** Lifespan was analyzed for control flies (*w^1118^*) and flies expressing *DptA* RNAi (*DptA* KD*,* **A**), *AttA* RNAi (*AttA* KD*,* **B**), *AttC* RNAi (*AttC* KD*,* **B**), *PGRP-SC1b* RNAi (*PGRP-SC1b* KD*,* **C**), *PGRP-LE* RNAi (*PGRP-LE* KD*,* **C**), *Tak1* RNAi (*Tak1* KD*,* **D**), and *Tak2* RNAi (*Tak2* KD*,* **D**)*.* The number of flies (n) is indicated in the figure. Log-rank test. *p < 0.05, n.s., no significance.

**(E)** A schematic illustration of Relish-related signaling pathway. Genes marked in red are implicated in muscle aging, while genes marked in black showed no apparent effect on muscle aging. Genes represented in grey were not included in the screen.


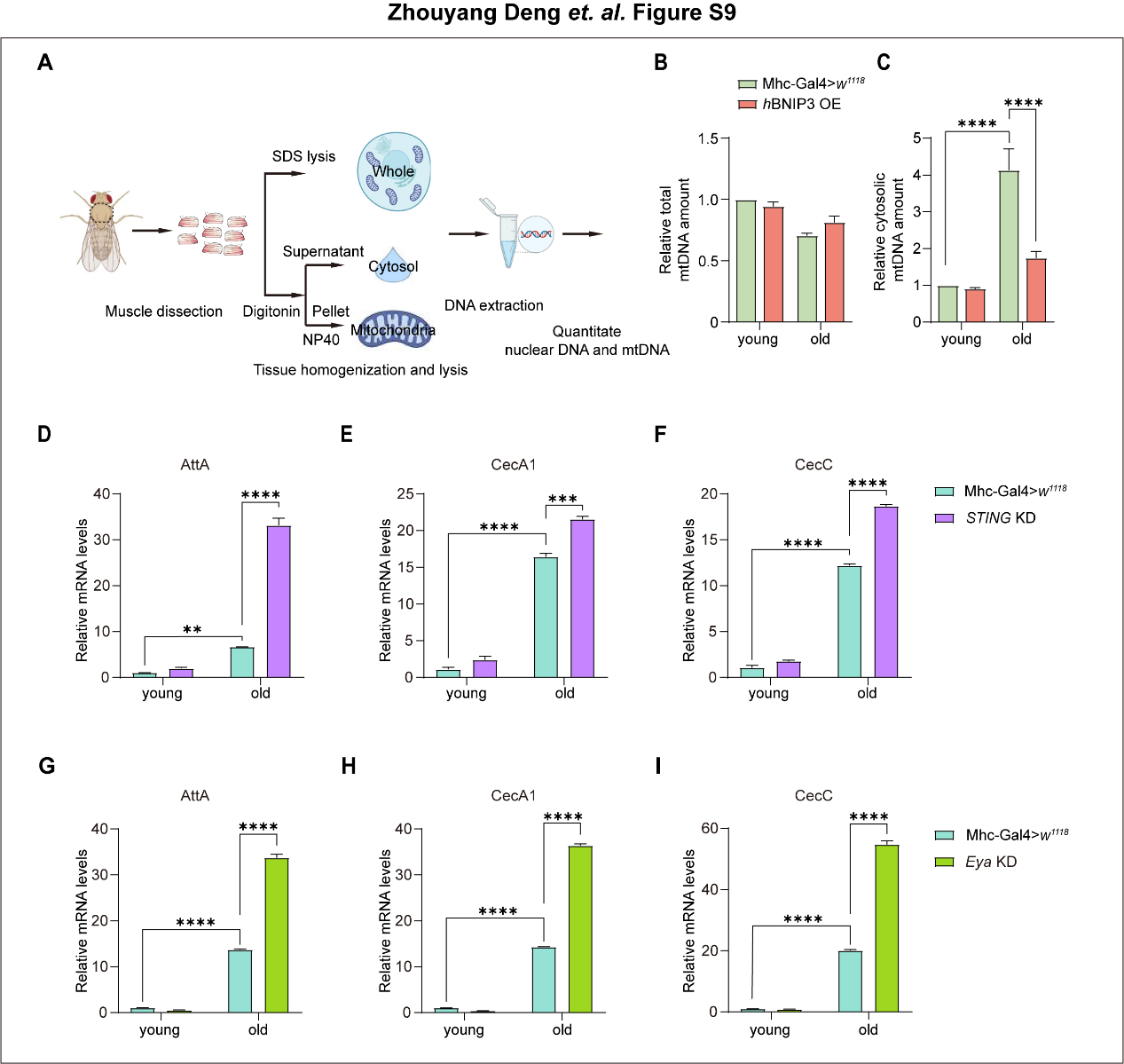


**Supplementary Figure S9. BNIP3 inhibits mtDNA release through a STING/Eya-independent mechanism in aging flies**

1. Flow chart of total and cytosolic mtDNA extraction from *Drosophila* muscle tissue.

**(B and C)** Total **(B)** and cytosolic **(C)** mtDNA levels in IFMs of control flies (Mhc-Gal4>*w^1118^*) and flies overexpressing *h*BNIP3 (*h*BNIP3 OE) were determined by quantitative PCR and normalized to the mean value of the young controls. Young, 5-day-old; old, 30-day-old. Two-way ANOVA followed by Tukey’s test. ****p < 0.0001. Data are from at least three biological replicates and presented as mean ± SEM. Note that cytosolic mtDNA is significantly increased in aging flies. The increase is suppressed by *h*BNIP3 expression.

**(D-F)** Aging-induced expression of AttA, CecA1 and CecC is further increased by *STING* knockdown. Thoraces derived from control flies (Mhc-Gal4>*w^1118^*) and flies with *STING* RNAi (*STING* KD) were analyzed by qPCR. Young, 5-day-old; old, 30-day-old. Two-way ANOVA followed by Tukey’s test. ****p < 0.0001, ***p < 0.001, **p < 0.01. Data are from at least three biological replicates and presented as mean ± SEM.

**(G-I)** Aging-induced expression of AttA, CecA1 and CecC is further increased by *Eya* knockdown. Thoraces derived from control flies (Mhc-Gal4>*w^1118^*) and flies with *Eya* RNAi (*Eya* KD) were analyzed by qPCR. Young, 5-day-old; old, 30-day-old. Two-way ANOVA followed by Tukey’s test. ****p < 0.0001. Data are from at least three biological replicates and presented as mean ± SEM.

**
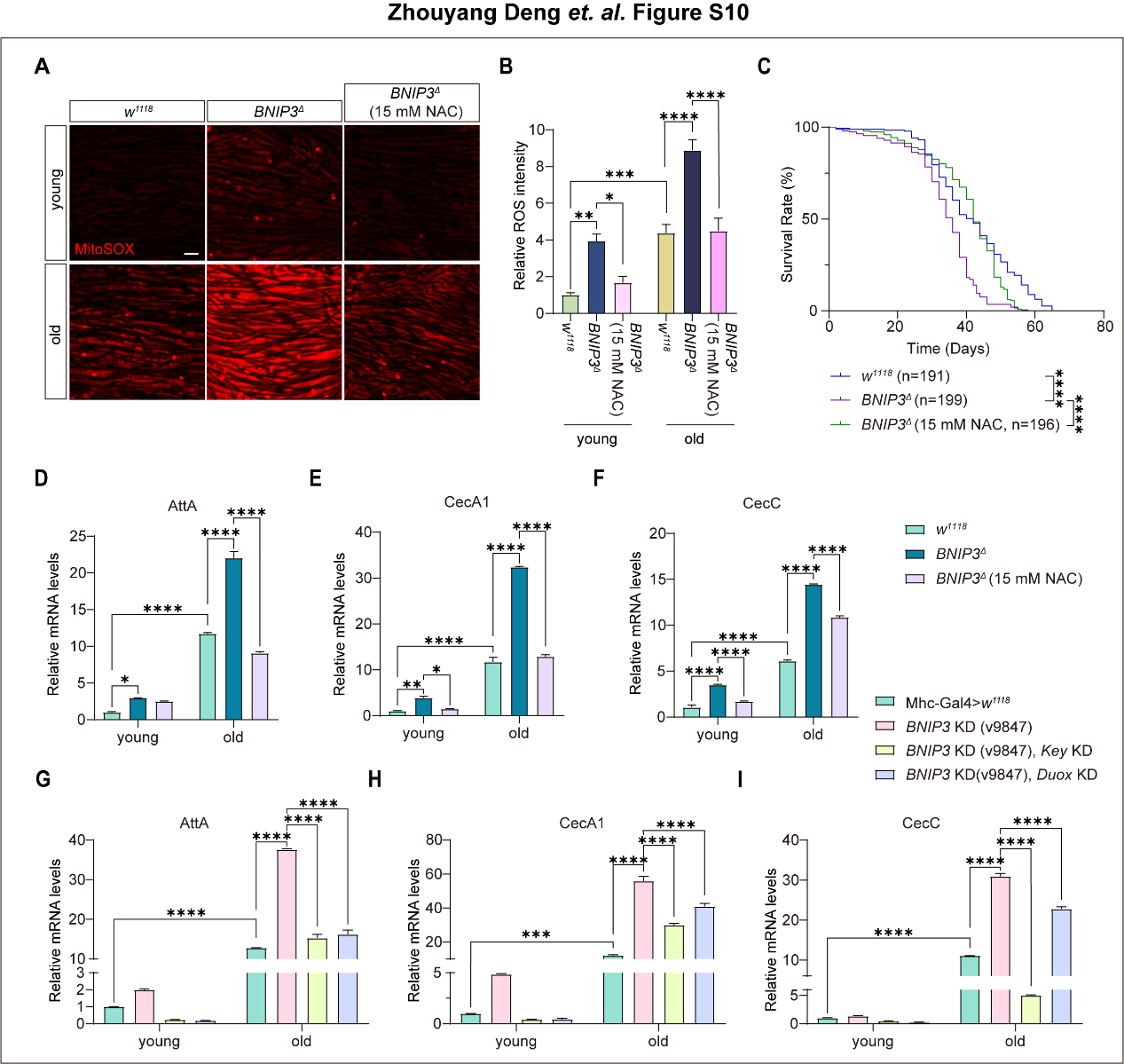
**

**Supplementary Figure S10. ROS clearance rescues the shortened lifespan of *BNIP3****^Δ^* **flies**

**(A and B)** BNIP3 deletion-induced ROS production is suppressed by NAC treatment. IFMs derived from young (5-day-old) and old (30-day-old) flies, including wildtype flies (*w^1118^*) and *BNIP3^Δ^* mutant flies, treated with or without 15 mM NAC were stained with MitoSOX. Mitochondrial superoxide was shown as red. Bar = 10 μm **(A)**. Quantification of ROS in IFMs. n = 6 IFMs for every group **(B)**. Two-way ANOVA followed by Tukey’s test. ****p < 0.0001, ***p < 0.001, **p < 0.01, *p < 0.05. Data are from at least three biological replicates and presented as mean ± SEM.

**(C)** NAC treatment rescued shortened lifespan of *BNIP3^Δ^* mutant flies. Wildtype flies (*w^1118^*) and *BNIP3^Δ^* mutant flies treated with or without 15 mM NAC were assayed for lifespan. n numbers were indicated in the figure. Log-rank test. ****p < 0.0001.

**(D-F)** Expression of AttA, CecA1 and CecC. Thoraces derived from young (5-day-old) and old (30-day-old) flies, including wildtype flies (*w^1118^*) and *BNIP3^Δ^* mutant flies, treated with or without 15 mM NAC were analyzed. Two-way ANOVA followed by Tukey’s test. ****p < 0.0001, ***p < 0.001, **p < 0.01, *p < 0.05. Data are presented as mean ± SEM from at least three biological replicates.

**(G-I)** Expression of AttA, CecA1 and CecC. Thoraces derived from young (5-day-old) and old (30-day-old) flies, including wildtype flies (Mhc-Gal4>*w^1118^*), flies with *BNIP3* knockdown (*BNIP3* KD, v9847), and flies expressing *BNIP3* RNAi together with either *Key* RNAi (*BNIP3* KD, *Key* KD) or *Duox* RNAi (*BNIP3* KD, *Duox* KD), were analyzed. Two-way ANOVA followed by Tukey’s test. ****p < 0.0001, ***p < 0.001. Data are from at least three biological replicates and presented as mean ± SEM.


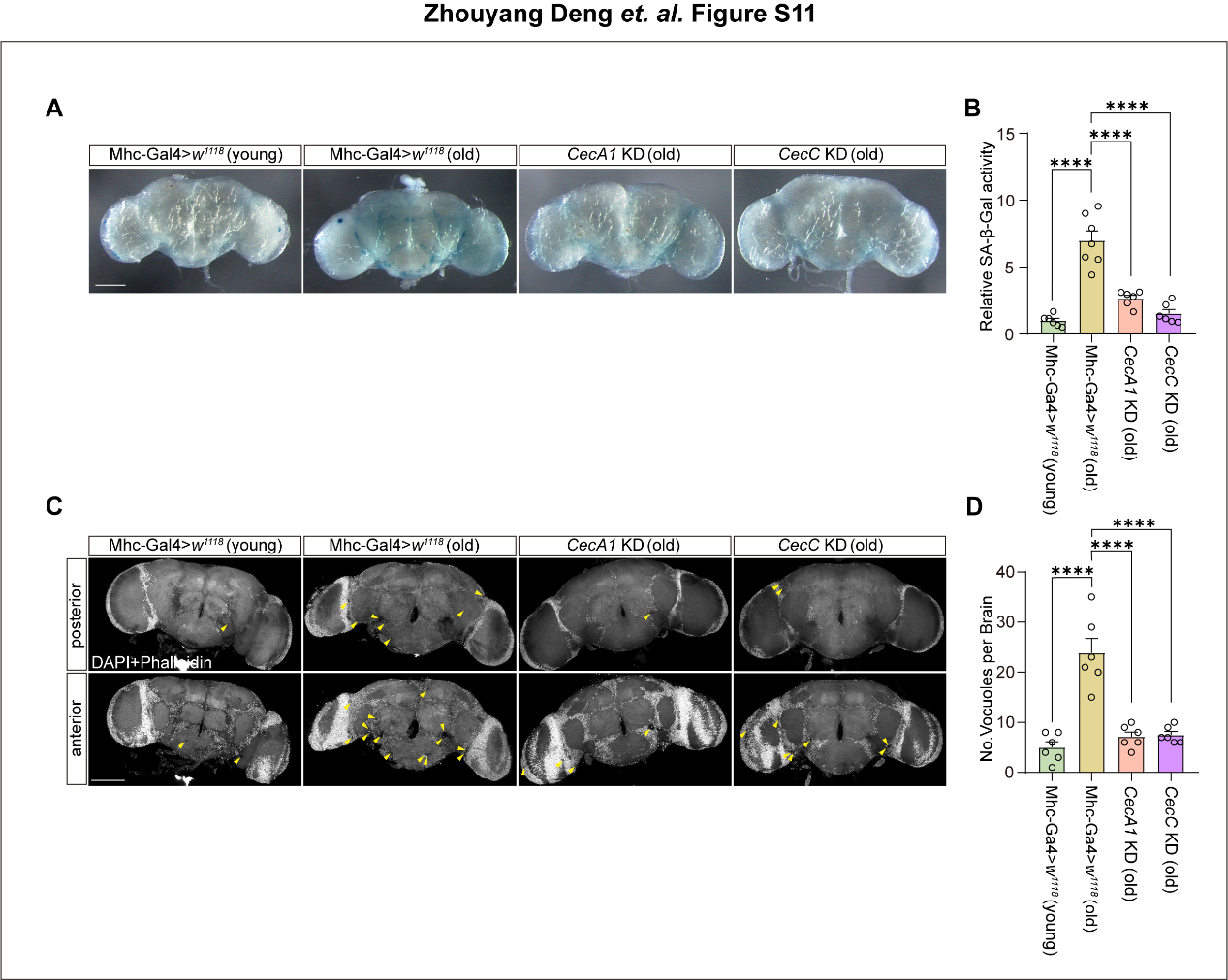


**Supplementary Figure S11. Knockdown of *CecA1* or *CecC* in skeletal muscle suppresses age-dependent neurodegeneration in *Drosophila***

**(A and B)** Knockdown of *CecA1* or *CecC* inhibits age-associated SA-β-Gal activity in fly brains. Brains from control flies (Mhc-Gal4>*w^1118^*) and flies with muscle-specific *CecA1* RNAi (*CecA1* KD) or *CecC* RNAi (*CecC* KD) were analyzed. Young, 5-day-old; old, 30-day-old. Bar = 100 μm **(A)**. Quantification of SA-β-Gal staining. n = 6 (Mhc-Gal4>*w^1118^*, young), 7 (Mhc-Gal4>*w^1118^*, old), 6 (*CecA1* KD, old) and 6 (*CecC* KD, old) **(B)**. One-way ANOVA followed by Tukey’s test. ****p < 0.0001. Data are from at least three biological replicates and presented as mean ± SEM.

**(C and D)** Knockdown of *CecA1* or *CecC* suppresses aging-related vacuole formation in fly brains. Brains from control flies (Mhc-Gal4>*w^1118^*) and flies with muscle-specific *CecA1* RNAi (*CecA1* KD), or *CecC* RNAi (*CecC* KD) were analyzed. Young, 5-day-old; old, 30-day-old. Yellow arrows indicate pathological degenerative vacuoles. Bar=100 μm **(C)**. Quantification of vacuoles. n = 6 brains per group were analyzed **(D)**. One-way ANOVA followed by Tukey’s test. ****p < 0.0001. Data are from at least three biological replicates and presented as mean ± SEM.

**Reference:**

Katayama, H., H. Hama, K. Nagasawa, H. Kurokawa, M. Sugiyama, R. Ando, M. Funata, N. Yoshida, M. Homma, T. Nishimura, M. Takahashi, Y. Ishida, H. Hioki, Y. Tsujihata, and A. Miyawaki. 2020. Visualizing and Modulating Mitophagy for Therapeutic Studies of Neurodegeneration. *Cell*. 181:1176-1187 e1116.
